# The Detection of Phase Amplitude Coupling during Sensory Processing

**DOI:** 10.3389/fnins.2017.00487

**Published:** 2017-09-01

**Authors:** Robert A. Seymour, Gina Rippon, Klaus Kessler

**Affiliations:** ^1^Aston Brain Centre, School of Life and Health Sciences, Aston University Birmingham, United Kingdom; ^2^Department of Cognitive Science, Macquarie University Sydney, NSW, Australia; ^3^ARC Centre of Excellence in Cognition and Its Disorders, Macquarie University Sydney, NSW, Australia

**Keywords:** PAC, phase, amplitude, sensory, oscillations, MEG, alpha, gamma

## Abstract

There is increasing interest in understanding how the phase and amplitude of distinct neural oscillations might interact to support dynamic communication within the brain. In particular, previous work has demonstrated a coupling between the phase of low frequency oscillations and the amplitude (or power) of high frequency oscillations during certain tasks, termed phase amplitude coupling (PAC). For instance, during visual processing in humans, PAC has been reliably observed between ongoing alpha (8–13 Hz) and gamma-band (>40 Hz) activity. However, the application of PAC metrics to electrophysiological data can be challenging due to numerous methodological issues and lack of coherent approaches within the field. Therefore, in this article we outline the various analysis steps involved in detecting PAC, using an openly available MEG dataset from 16 participants performing an interactive visual task. Firstly, we localized gamma and alpha-band power using the Fieldtrip toolbox, and extracted time courses from area V1, defined using a multimodal parcelation scheme. These V1 responses were analyzed for changes in alpha-gamma PAC, using four common algorithms. Results showed an increase in alpha (7–13 Hz)–gamma (40–100 Hz) PAC in response to the visual grating stimulus, though specific patterns of coupling were somewhat dependent upon the algorithm employed. Additionally, *post-hoc* analyses showed that these results were not driven by the presence of non-sinusoidal oscillations, and that trial length was sufficient to obtain reliable PAC estimates. Finally, throughout the article, methodological issues and practical guidelines for ongoing PAC research will be discussed.

## Introduction

Electrophysiological brain oscillations are often separated into distinct frequency bands, ranging from low-frequency delta (1–4 Hz) to high-frequency gamma (<40 Hz). The power and/or connectivity profiles of these frequency bands have been linked with specific neuronal and cognitive functions (Buzsáki and Draguhn, 2004; Palva et al., 2005). Whilst this has proven a powerful tool in neuroscientific research, there is emerging evidence that oscillations from different frequency bands also display specific coupling patterns—a phenomenon termed cross frequency coupling (CFC; Jensen and Colgin, 2007; Hyafil et al., 2015). One of the best studied forms of CFC is phase-amplitude coupling (PAC), in which the amplitude/power of a high frequency oscillation, often gamma (>40 Hz), is coupled to the phase of a lower frequency oscillation (Canolty et al., 2006; Canolty and Knight, 2010). PAC has been observed in multiple regions of the human brain, including the visual cortex (Voytek et al., 2010), auditory cortex (Cho et al., 2015), hippocampus (Lega et al., 2014; Heusser et al., 2016), and prefrontal cortex (Voloh et al., 2015; Voytek et al., 2015), in both electrocorticography (ECOG) and magnetoencephalography (MEG) recordings.

Within the visual system, there is strong evidence for a dynamic coupling between alpha phase (8–13 Hz) and gamma amplitude (>40 Hz; Voytek et al., 2010; Spaak et al., 2012; Bonnefond and Jensen, 2015). Alpha oscillations are associated with pulses of cortical inhibition every ~100 ms (Jensen and Mazaheri, 2010; Klimesch, 2012), whilst supporting communication through phase dynamics (Fries, 2015). In contrast, gamma oscillations emerge through local excitatory and inhibitory interactions, and synchronize local patterns of cortical activity (Singer and Gray, 1995; Buzsáki and Wang, 2012). In visual cortex, ongoing gamma-band activity becomes temporally segmented by distinct phases of alpha-band activity (Spaak et al., 2012; Bonnefond et al., 2017), possibly via inter-laminar coupling between supragranular and infragranular cortical layers (Mejias et al., 2016). Intriguingly, this coupling has been proposed to act as a mechanism for the dynamic co-ordination of brain activity over multiple spatial scales, with high-frequency activity within local ensembles coupled to large-scale patterns of low-frequency phase synchrony (Bonnefond et al., 2017), both within the visual system (Bonnefond and Jensen, 2015), and more widespread neurocognitive networks (Florin and Baillet, 2015). This would allow information to be routed efficiently between areas and for neuronal representations to be segmented and maintained, for example during visual working memory (Lisman and Idiart, 1995; Bonnefond and Jensen, 2015). Atypical patterns of PAC have also been proposed to underlie atypical cortical connectivity in several neurological conditions, including autism spectrum disorder (Khan et al., 2013; Kessler et al., 2016), schizophrenia (Kirihara et al., 2012), and Parkinson's Disease (Özkurt and Schnitzler, 2011; De Hemptinne et al., 2013).

Given the developing interest in cross-frequency coupling, it is vital for the wider neuroscience and electrophysiological community to understand the steps involved in its measurement and interpretation. This is especially important for PAC, which is beset with methodological pitfalls, since there are many competing algorithms, approaches, and currently no gold-standard set of analysis steps (Canolty and Knight, 2010; Jensen et al., 2016). It has also been suggested that numerous incidences of reported PAC may in fact be false positives, caused by suboptimal analysis practices and/or the presence of artifacts within the data (Aru et al., 2015; Hyafil, 2015). For example non-sinusoidal sawtooth-like oscillations can generate artificially inflated PAC values, via low-frequency phase harmonics (Lozano-Soldevilla et al., 2016; Cole et al., 2017; Vaz et al., 2017).

In this article, we outline a general approach for detecting changes in phase-amplitude coupling during visual processing, using a novel MEG dataset, analyzed using the Fieldtrip toolbox (Oostenveld et al., 2010), and openly available MATLAB scripts. Four common PAC algorithms were used to quantify the coupling between ongoing alpha phase (7–13 Hz) and gamma amplitude/power (>40 Hz) whilst participants viewed a static visual grating. Given the controversy surrounding PAC analysis, methodological steps were outlined in detail and justified by existing empirical research. Furthermore, follow-up analyses were conducted to establish the reliability of our results and to assess whether patterns of alpha-gamma PAC were driven by non-sinusoidal oscillations or insufficient data.

## Methods

### Participants

Data were collected from 16 participants (6 male, 10 female, mean age = 28.25, *SD* = 6.23). All participants had normal or corrected to normal vision and no history of neurological or psychiatric illness.

### Experimental procedures

All experimental procedures complied with the Declaration of Helsinki and were approved by the Aston University, Department of Life and Health Sciences ethics committee. Participants gave written informed consent before participating in the study.

### Paradigm

**Figure 1:**
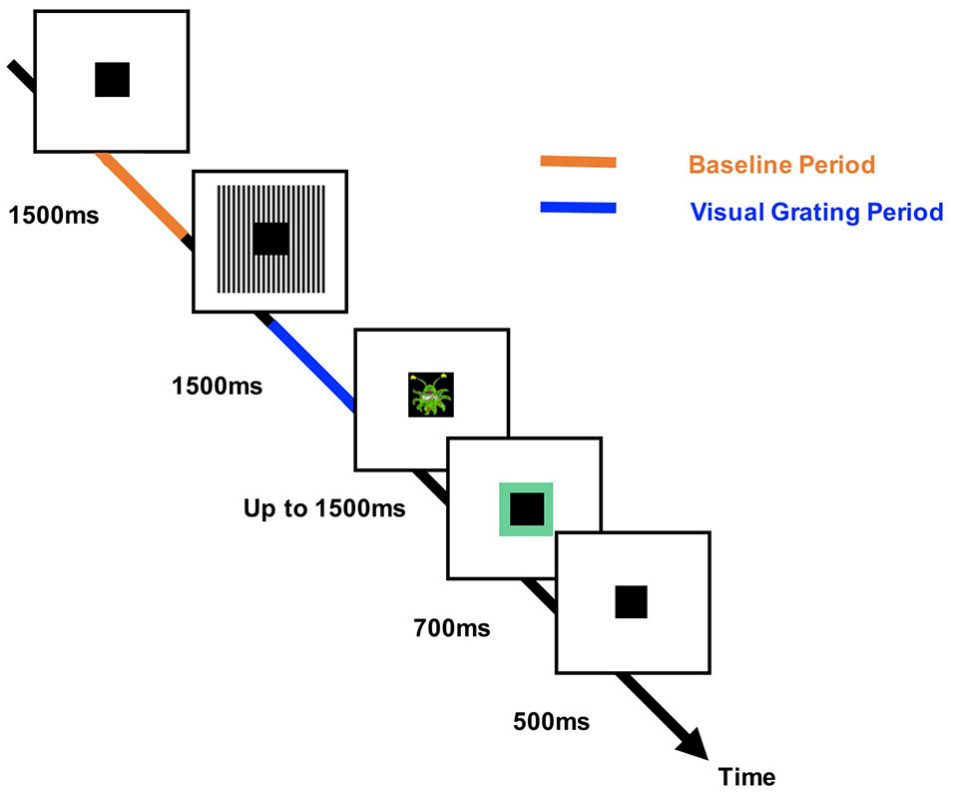
The structure of the engaging sensory paradigm. Each trial started with a 1,500, 2,500, or 3,500 ms baseline period in which a square black box (the “porthole”) was centrally presented. This was followed by presentation of the visual grating stimulus (two cycles/degree) around the central porthole for 1,500 ms. A picture of an alien (target) or astronaut (non-target) was then shown within the porthole for 500 ms. Participants were instructed to respond after the appearance of an alien picture (maximum response time: 1,500 ms). Correct or incorrect responses were conveyed to the participant through audio-visual feedback in which the porthole turned green (correct) or red (incorrect) and a correct/incorrect tone was played. The times corresponding to the analyzed baseline and visual grating periods are labeled in orange/blue, respectively.

Participants performed an engaging sensory paradigm (Figure 1), designed to elicit patterns of high-frequency oscillatory activity. Each trial started with a variable fixation period of 1,500, 2,500, or 3,500 ms randomized across trials, followed by the presentation of a visual grating or auditory binaural click train stimulus; however only the visual data will be analyzed in this article. The visual grating stimulus had a spatial frequency of two cycles/degree and was presented for 1,500 ms. To keep participants engaged with the task, cartoon pictures of aliens or astronauts were presented after the visual grating, for a maximum of 500 ms. Please note that visual responses to the alien or astronaut picture did not form part of the MEG analysis. Participants were instructed to respond to the appearance of an alien picture using a response pad (maximum response period of 1,500 ms). The accuracy of the response was conveyed through audio-visual feedback, followed by a 500 ms fixation period. In total, the MEG recording lasted 12–13 min and included 64 trials with visual grating stimuli. Prior to MEG acquisition, the nature of the task was fully explained to participants and several practice trials were performed. Accuracy rates were above 95% for all participants indicating that the task was engaging and successfully understood.

### MEG acquisition

MEG data were acquired using a 306-channel Neuromag MEG scanner (Vectorview, Elekta, Finland) made up of 102 triplets of two orthogonal planar gradiometers and one magnetometer. All recordings were performed inside a magnetically shielded room at a sampling rate of 1,000 Hz. Five head position indicator (HPI) coils were applied for continuous head position tracking, and visualized post-acquisition using an in-house Matlab script. For MEG-MRI coregistration purposes three fiducial points, the locations of the HPI coils and 300–500 points from the head surface were acquired using the integrated Polhemus Fastrak digitizer.

Visual stimuli were presented on a screen located 86 cm from participants (resulting in two cycles/degree for the visual grating), and auditory feedback through MEG-compatible earphones.

### Structural MRI

A structural T1 brain scan was acquired for source reconstruction using a Siemens MAGNETOM Trio 3T scanner with a 32-channel head coil (TE = 2.18 ms, TR = 2,300 ms, TI = 1,100 ms, flip angle = 9°, 192 or 208 slices depending on head size, voxel-size = 0.8 × 0.8 × 0.8 cm).

### MEG-MRI coregistration and 3D cortical mesh construction

MEG data were co-registered with participants MRI structural scan by matching the digitized head shape data with surface data from the structural scan (Jenkinson and Smith, 2001). The aligned MRI-MEG image was used to create a forward model based on a single-shell description of the inner surface of the skull (Nolte, 2003), using the segmentation function in SPM8 (Litvak et al., 2011). The cortical mantle was then extracted to create a 3D cortical mesh, using Freesurfer v5.3 (Fischl, 2012), and registered to a standard fs_LR mesh, based on the Conte69 brain (Van Essen, 2012), using an interpolation algorithm from the Human Connectome Project (Van Essen et al., 2012; instructions here: https://goo.gl/3HYA3L). Finally, the mesh was downsampled to 4002 vertices per hemisphere. Due to the extensive computation time involved in these procedures, all participant-specific cortical meshes are available to download in the /anat directory of the Figshare repository (see later).

### Pre-processing

MEG data were pre-processed using Maxfilter (temporal signal space separation, .9 correlation), which suppresses external sources of noise from outside the head (Taulu and Simola, 2006).

Further pre-processing steps were performed in Matlab 2014b using the open-source Fieldtrip toolbox v20161024 (Oostenveld et al., 2010; script: 1_preprocessing_elektra_frontiers_PAC.m). Firstly, for each participant the entire recording was band-pass filtered between 0.5 and 250 Hz (Butterworth filter, low-pass order 4, high-pass order 3) and band-stop filtered (49.5–50.5 Hz; 99.5–100.5 Hz) to remove residual 50 Hz power-line contamination and its harmonics. Data were then epoched into segments of 4,000 ms (1,500 ms pre, 1,500 ms post-stimulus onset, with 500 ms of padding either side) and each trial was demeaned and detrended. Trials containing artifacts (SQUID jumps, eye-blinks, head movement, muscle) were removed if the trial-by-channel (magnetomer) variance exceeded 8 × 10^−23^, resulting in an average of 63.5 trials per condition, per participant. Indices of removed trials are included in the Supplementary Materials. Site-specific MEG channels containing large amounts of non-physiological noise were removed from all analyses (MEG channels: 0111, 0322, 2542, 0532).

### Source analysis

Source analysis was conducted using a linearly constrained minimum variance beamformer (LCMV; Van Veen et al., 1997), which applies a spatial filter to the MEG data at each vertex of the 3D cortical mesh, in order to maximize signal from that location whilst attenuating signals elsewhere. Beamforming weights were calculated by combining the covariance matrix of the sensor data with leadfield information. Due to rank reduction following data cleaning with Maxfilter, the covariance matrix was kept at a rank below 64 components, which explained 99% of the variance. For all analyses, a common filter was used across baseline and grating periods, and a regularization parameter of lambda 5% was applied.

Due to prior interest in the gamma and alpha-bands (Hoogenboom et al., 2006; Muthukumaraswamy et al., 2010; Michalareas et al., 2016), the visual data were band-pass filtered (Butterworth filter) between 40–60 Hz (gamma) and 8–13 Hz (alpha), and source analysis was performed separately for each frequency band. To capture induced rather than evoked visual activity, a period of 300–1,500 ms following stimulus onset was compared with a 1,200 ms baseline period (1,500–300 ms before grating onset). The change in oscillatory power for each vertex was averaged across participants, interpolated onto a 3D mesh provided by the Human Connectome Project (Van Essen, 2012), and thresholded at a value which allowed the prominent patterns of power changes to be determined (see **Figure 3**, script: 2_get_source_power.m).

### Extracting area V1 time-series

Trial time-courses were extracted from bilateral visual area V1, defined using a multi-modal parcelation from the Human Connectome Project, which combined retinotopic mapping, T1/T2 structural MRI and diffusion-weighted MRI to accurately define the boundaries between cortical areas (Glasser et al., 2016; **Figure 3C**). The downsampled version of this atlas can be found in the parent directory of the Figshare repository (see later). To obtain a single spatial filter from this region, we performed a principle components analysis (PCA) on the concatenated filters from 182 vertices of bilateral V1, multiplied by the sensor-level covariance matrix, and extracted the first component. The sensor-level data was then multiplied by this spatial filter to obtain a V1-specific “virtual electrode” (script: 3_get_VE_frontiers_PAC.m), and the change in oscillatory power between grating and baseline periods was calculated from 1 to 100 Hz, using a 500 ms time window, sliding in steps of 20 ms and ±8 Hz frequency smoothing (script: 4_calc_pow_change.m). It is important to note that while we decided to use a multimodal atlas, visual area V1 virtual electrode time-series could also be defined using a more standard volumetric approach, for example the AAL atlas, which is included in the Fieldtrip toolbox (Oostenveld et al., 2010).

### Phase amplitude coupling (PAC) analysis

**Figure 2:**
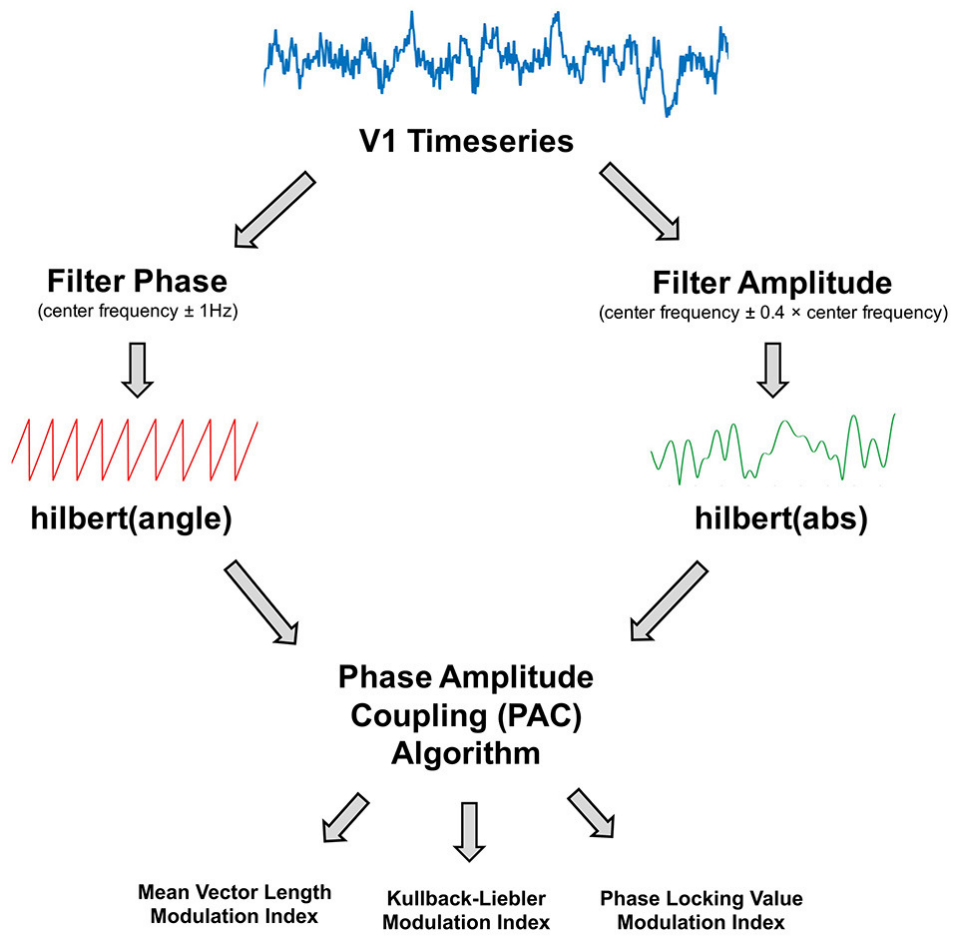
Illustration of the phase amplitude coupling (PAC) analysis procedure. The V1 time-series were filtered to obtain estimates of phase and amplitude, using a narrow (±1 Hz) bandwidth for the phase and a variable bandwidth (±0.4 times the center frequency) for the amplitude. Phase and amplitude information were obtained via the Hilbert transform. The coupling between phase and amplitude was then quantified using Mean Vector Length, Kullback-Leiber, or Phase Locking Value algorithms to produce a Modulation Index value.

V1 time-courses were examined for changes in alpha-gamma phase amplitude coupling (PAC). The general procedure is outlined in Figure 2. The first step was to obtain estimates of low frequency phase (*f*_p_) and high frequency amplitude (*f*_a_) for each trial using a fourth order, two-pass Butterworth filter, and then applying the Hilbert transform (Le Van Quyen et al., 2001). To avoid sharp edge artifacts, which can result in spurious PAC (Kramer et al., 2008), the first 500 ms and last 500 ms of each trial was discarded.

The bandwidth of the filter used to obtain *f*_p_ and *f*_a_ is a crucial parameter in calculating PAC (Aru et al., 2015). The filters for extracting *f*_a_ need to be wide enough to capture the center frequency ± the modulating *f*_p_. So, for example, to detect PAC between *f*_p_ = 13 Hz and *f*_a_ = 60 Hz, requires a *f*_a_ bandwidth of at least 13 Hz [47 73]. If this condition is not met, then PAC cannot be detected even if present (Dvorak and Fenton, 2014). We therefore decided to use a variable bandwidth, defined as ±0.4 times the center frequency (e.g., for an amplitude of 60 Hz, the bandwidth was 24 Hz either side [36 84]), which has been shown to improve the ability to detect PAC (Berman et al., 2012; Voloh et al., 2015). For alpha-band phase (maximum 13 Hz), this allowed us to calculate PAC for amplitudes above 34 Hz. The bandwidth for *f*_p_ was kept narrow (1 Hz ± the center frequency), in order to extract sinusoidal waveforms. Furthermore, each trial was visually inspected to confirm that the *f*_p_ filtered oscillations were sinusoidal in nature.

Next, the coupling between *f*_p_ and *f*_a_ was quantified using four common PAC approaches^1^: the Mean-Vector Length modulation index, originally described in Canolty et al. (2006); the Mean-Vector Length modulation index described in Özkurt and Schnitzler (2011); the phase-locking value modulation index described in Cohen (2008); and the Kullback-Lieber modulation index described in Tort et al. (2010b). These approaches were selected due to their popularity in the MEG/EEG PAC literature (e.g., Mathewson et al., 2011; Khan et al., 2013; Bonnefond and Jensen, 2015; Cho et al., 2015), and to demonstrate the diversity of PAC results based on the algorithm selected.

The mean vector length modulation index (MVL-MI-Canolty) approach estimates PAC from a signal with length N, by combining phase (ϕ) and amplitude information to create a complex-valued signal: *f_a_*^*e*^*i*(ϕ*f_p_*)^^ (Canolty et al., 2006), in which each vector corresponds to a certain time-point (*n*). If the resulting probability distribution function is non-uniform, this suggests a coupling between *f*_p_ and *f*_a_, which can be quantified by taking the length of the average vector.

$$MI=\left|\frac{1}{N}\sum_{n=1}^{N}{\text{f}}_{a}\left(n\right)e^{i\left(\phi {\text{f}}_{p}\left(n\right)\right)}\right|$$

However, MI-values from the MVL-MI-Canolty algorithm have been shown to partly reflect the power of *f*_a_ oscillations, rather than their coupling (Canolty and Knight, 2010). Therefore, as an alternative to surrogate data, we applied a MVL-MI algorithm from Özkurt and Schnitzler (2011), which includes a normalization factor corresponding to the power of *f*_a_. Özkurt and Schnitzler (2011) suggest that their algorithm is more resilient to measurement noise, and is therefore highly relevant for MEG data, which has an inherently lower signal-to-noise ratio compared with invasive electrophysiological recordings (Goldenholz et al., 2009).

$$MI=\frac{1}{\sqrt{N}}\frac{\left|\frac{1}{N}\sum_{n=1}^{N}{\text{f}}_{a}\left(n\right)e^{i\left(\phi {\text{f}}_{p}\left(n\right)\right)}\right|}{\sqrt{\frac{1}{N}\sum_{n=1}^{N}{\text{f}}_{a}{\left(n\right)}^{2}}}$$

The PLV-MI-Cohen approach assumes that if PAC is present, the envelope of *f*_a_ should oscillate at the frequency corresponding to *f*_p_. The phase of *f*_a_ envelope can be obtained by applying the Hilbert transform (angle): ϕ*f*_a_. The coupling between the low-frequency ϕ*f*_p_ phase values and the phase of the amplitude envelope, ϕ*f*_a_, can be quantified by calculating a phase locking value (PLV), in much the same way as determining phase synchronization between electrophysiological signals.

$$MI=\left|\frac{1}{N}\sum_{n=1}^{N}e^{i(\phi {\text{f}}_{p}\left(n\right)-\phi {\text{f}}_{a}\left(n\right)}\right|$$

Finally, the KL-MI-Tort approach estimates PAC by quantifying the amount of deviation in amplitude-phase distributions. This involves breaking *f*_p_ into 18 bins, and calculating the mean amplitude within each phase bin, normalized by the average value across all bins. Although the number of phase bins chosen is arbitrary, the specific number (9, 18, or 36) does not seem to influence PAC estimation (Figure S1; van Driel et al., 2015). The modulation index is calculated by comparing the amplitude-phase distribution (*P*) against the null hypothesis of a uniformly amplitude-phase distribution (*Q*).

$$MI=\frac{D\left(P,Q\right)}{\log\left(N_{bins}\right)}$$

Mathematically, this is computed using the Kullbeck-Leiber distance (*D*), related to Shannon's entropy.

$$D\left(P,Q\right)=\sum_{i_{bin}=1}^{N}P(i_{bin})\;\cdot \;\log\left(\frac{P\left(i_{bin}\right)}{Q\left(i_{bin}\right)}\right)$$

Using these four approaches (MVL-MI-Canolty; MVL-MI-Özkurt; KL-MI-Tort; PLV-MI-Cohen) we calculated PAC between phases 7–13 Hz (in 1 Hz steps) and amplitudes 34–100 Hz (in 2 Hz steps), for the time-period 300–1,500 ms following grating presentation and a 1,200 ms baseline period. PAC-values were calculated separately for each trial and then averaged to obtain a single MI-value per amplitude and phase. This was repeated using surrogate data, created by shuffling trial and phase-carrying information (200 surrogates), to normalize MI-values. On a PC with 32 GB of RAM, and Intel(R) Core™ i7-4790 processor, the computation time for these procedures was 4.5 h (script: 5_visual_PAC_four_methods.m).

To assess changes in the strength of PAC between the grating and baseline periods, the comodulograms were compared using non-parametric cluster-based statistics, which have been shown to adequately control the type-I error rate for electrophysiological data (Maris and Oostenveld, 2007). First, an uncorrected dependent-samples *t*-test was performed (grating vs. baseline), and all MI-values exceeding a 5% significance threshold were grouped into clusters. The maximum *t*-value within each cluster was carried forward. Next, a null distribution was obtained by randomizing the condition label (grating/baseline) 1,000 times and calculating the largest cluster-level *t*-value for each permutation. The maximum *t*-value within each original cluster was then compared against this null distribution, with values exceeding a threshold of *p* < 0.05 deemed significant.

### Sinusoidal oscillations

One major issue in cross-frequency coupling analysis is the presence of non-sinusoidal sawtooth-like oscillations (Jensen et al., 2016; Cole et al., 2017), which can result in spurious estimates of PAC (Lozano-Soldevilla et al., 2016). This property of oscillations can be quantified by calculating the time taken from trough to peak (rise-time), peak to trough (decay-time), and the ratio between these values (Dvorak and Fenton, 2014; Cole and Voytek, 2017). We therefore calculated this ratio for the visual V1 data from 7 to 13 Hz, and performed a *t*-test to check for differences in non-sinusoidal oscillations between grating and baseline periods (script: 6_check_non_sinusoidal.m).

### Simulated PAC analysis

To investigate the validity of the four PAC approaches, we constructed 1.2 s of simulated data with known alpha-gamma PAC [*f*_p_ = 10 Hz; *f*_a_ = 50–70 Hz; code adapted from Kramer et al. (2008) and Özkurt and Schnitzler (2011)] and added a random level of noise (signal-to-noise ratio >−11.5 dB). Comodulograms were produced using the four PAC algorithms on 64 trials of simulated data. Using the same code, we also investigated how the four algorithms were affected by trial length (0.1–10 s in 0.1 s steps, script: 7_simulated_PAC_analysis.m).

### Analysis code and data sharing

MEG data are available to download online at Figshare (https://doi.org/10.6084/m9.figshare.c.3819106.v1), along with participant-specific 3D cortical meshes. Access to the raw structural MRI data will be granted upon reasonable request and ethical approval from Aston University Life and Health Sciences ethics committee. Data analysis code has been made available to download from Figshare (permanent version: https://doi.org/10.6084/m9.figshare.5297032), Github (https://github.com/neurofractal/sensory_PAC) and within the Supplementary Materials. This includes MATLAB code for the four PAC algorithms, which can be applied to electrophysiological data arranged in the standard Fieldtrip format (Oostenveld et al., 2010). Please note that these scripts have been optimized for the Windows operating system and MATLAB versions above 2014b (see supplementary materials for other software dependencies). Successful use of the scripts requires the user to have at least a basic understanding of MATLAB, signal processing, and the methodological complexities surrounding PAC. We therefore direct the reader to a number of excellent reviews and empirical papers (Canolty et al., 2006; Jensen and Colgin, 2007; Canolty and Knight, 2010; Aru et al., 2015; Hyafil et al., 2015).

## Results

### Source localization

**Figure 3:**
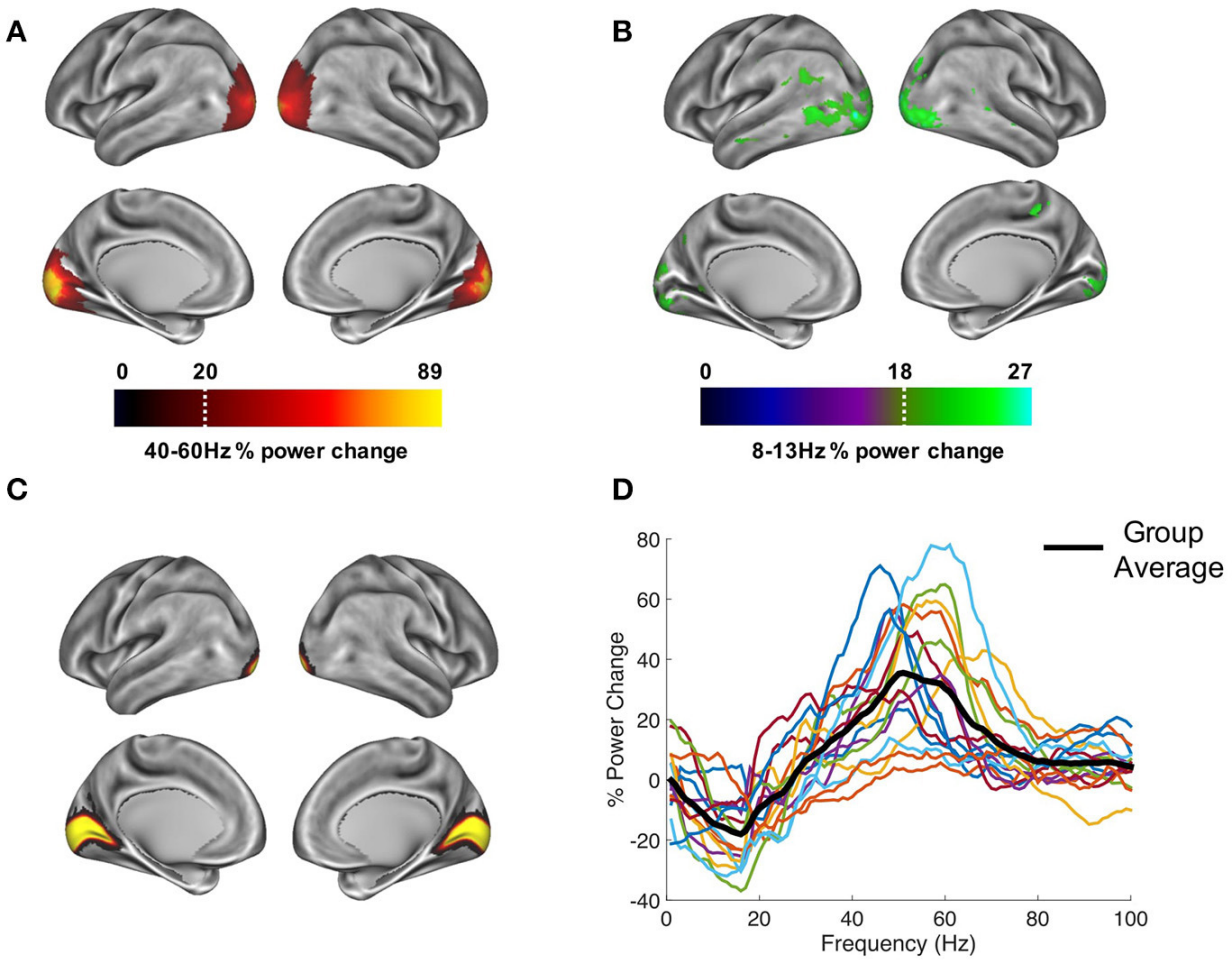
Whole-brain oscillatory power changes following the presentation of the visual grating are marked by **(A)** increases in the gamma-band (40–60 Hz) and **(B)** decreases in the alpha-band (8–13 Hz), localized primarily in the ventral occipital cortex. Power maps were thresholded at a value which allowed prominent patterns of power changes to be determined, indicated by the white dotted line. Time-courses were extracted from bilateral visual area V1, defined using the atlas region shown in **(C)** from the HCP-MMP 1.0 parcelation (Glasser et al., 2016). **(D)** These V1 responses showed reductions in alpha/beta power and increases in gamma-band (40–70 Hz) power.

In order to establish patterns of oscillatory power changes following presentation of the visual grating, gamma-band (40–60 Hz), and alpha-band power (8–13 Hz) were localized for a 300–1,500 ms period post-stimulus presentation. Results for the gamma-band (Figure 3A), show an increase in oscillatory power which localizes to the ventral occipital cortex (Hoogenboom et al., 2006). Results for the alpha band (Figure 3B) showed a general decrease in power, located primarily in occipital areas, but extending into temporal and parietal regions. The more widespread spatial pattern could reflect on-going upstream processes triggered by the appearance of the grating, for example anticipation of the upcoming target (Stenner et al., 2014).

### Visual area V1 power changes

Time courses from area V1 were extracted (Figure 3C), and the change in oscillatory power between grating and baseline periods from 1 to 100 Hz was calculated (Figure 3D). Whilst results show individual variability in peak frequencies and the strength of oscillatory power, on average, activity within visual area V1 displays a reduction in alpha/beta power (8–20 Hz), and an increase in gamma power (40–70 Hz). The MEG data, therefore display well-established patterns of alpha and gamma-band event-related synchronization and desynchronization within visual area V1 (Hoogenboom et al., 2006; Bonnefond and Jensen, 2015; Michalareas et al., 2016), which is a crucial first step in calculating reliable estimates of PAC (Aru et al., 2015).

### Alpha-gamma PAC

Visual area V1 responses were next examined for changes in alpha-gamma PAC. Specifically, we set out to test whether the coupling between alpha-band phase and gamma-band amplitude was altered during presentation of the visual grating. Phase-amplitude comodulograms were produced between a range of phase frequencies (7–13 Hz) and amplitude frequencies (34–100 Hz), using the four algorithms described in Section Methods: MVL-MI-Canolty; MVL-MI-Özkurt; PLV-MI-Cohen, and KL-MI-Tort. Grating and baseline comodulograms were compared using cluster-based non-parametric statistics (Maris and Oostenveld, 2007).

**Figure 4:**
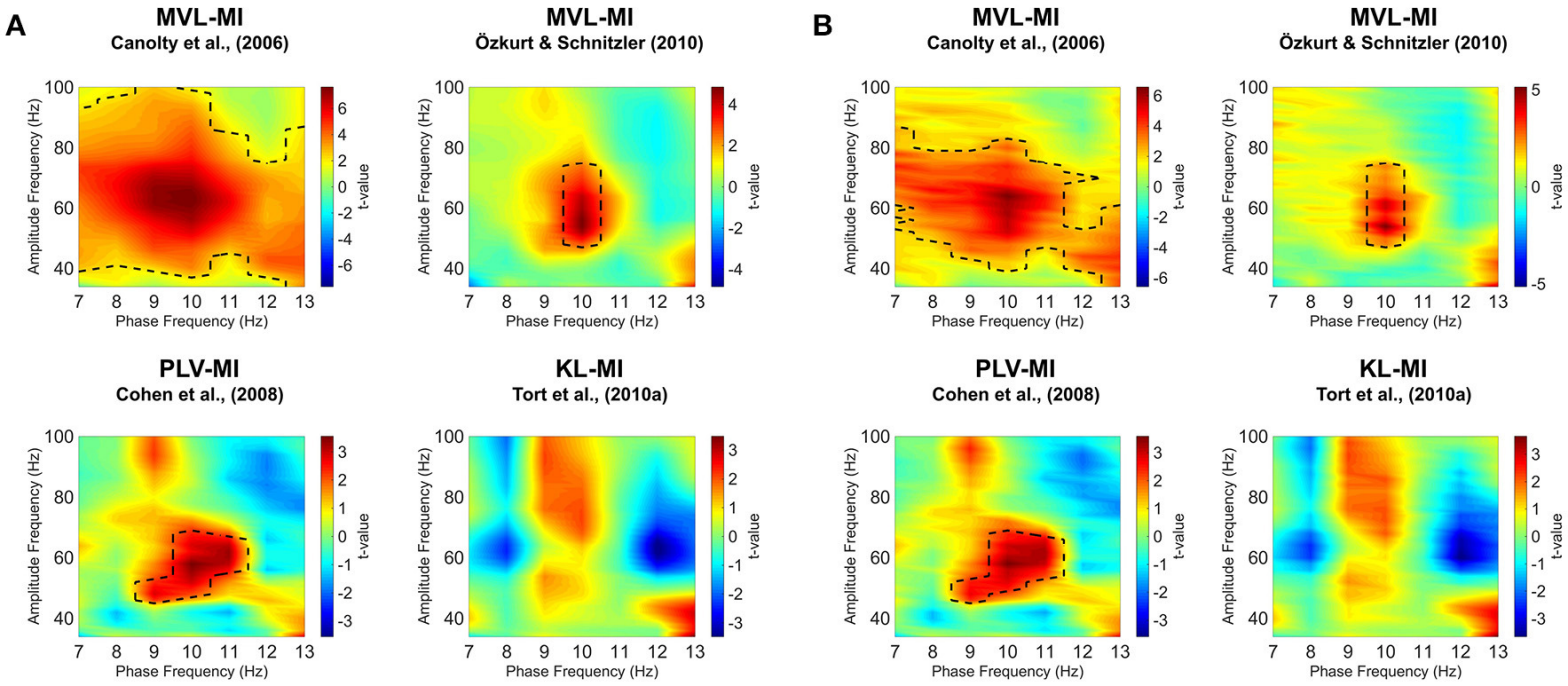
Phase-amplitude comodulograms produced by statistically comparing modulation index (MI)-values from 300 to 1,500 ms post-grating onset to a 1,200 ms baseline period, using four separate approaches. Comodulograms for **(A)** raw MI values and **(B)** MI values normalized by surrogate data are shown separately. The black dotted line represents significantly different phase-amplitude coupling frequencies (*p* < 0.05; for details of non-parametric cluster-based statistics see Section Methods).

Results are shown in Figure 4A. Using the MVL-MI-Canolty algorithm, there was a significant increase in alpha-gamma PAC over a large proportion of the comodulogram, between 40–100 Hz and 7–13 Hz, with a peak at 50–70 Hz amplitude and 9–10 Hz phase. This large area of significantly increased PAC is likely to reflect, in part, power increases in the gamma-band (Canolty et al., 2006). The alternative MVL-MI- Özkurt algorithm, which normalizes MI-values by the high-frequency oscillatory power, displayed a smaller area of significant coupling, with increased PAC between an amplitude of 50–70 Hz and phase of 10 Hz. There was also a similar cluster of significantly increased PAC between 9–11 Hz and 50–70 Hz using the PLV-MI-Cohen approach. The KL-MI-Tort results showed clusters of increased PAC between amplitudes of 50–100 Hz and phases of 9–10 Hz, but decreased PAC between amplitudes of 60–90 Hz and phases of 12–13 Hz. However, none of these clusters passed a significance threshold of *p* < 0.05 (two-tailed). Similar results were obtained after normalizing MI values with surrogate data (Figure 4B).

### Non-sinusoidal oscillations

To determine whether our alpha-gamma PAC results were driven by differences in the sinusoidal properties of oscillations between baseline and grating periods, the ratio between oscillatory rise-time and decay-time was calculated. For the alpha phase frequencies (7–13 Hz), there was no difference in this ratio (all frequencies *p* > 0.05), suggesting that our results are unlikely to be caused by increased non-sinusoidal sawtooth-like properties of alpha oscillations during stimulus period compared to baseline.

### Simulated PAC

**Figure 5:**
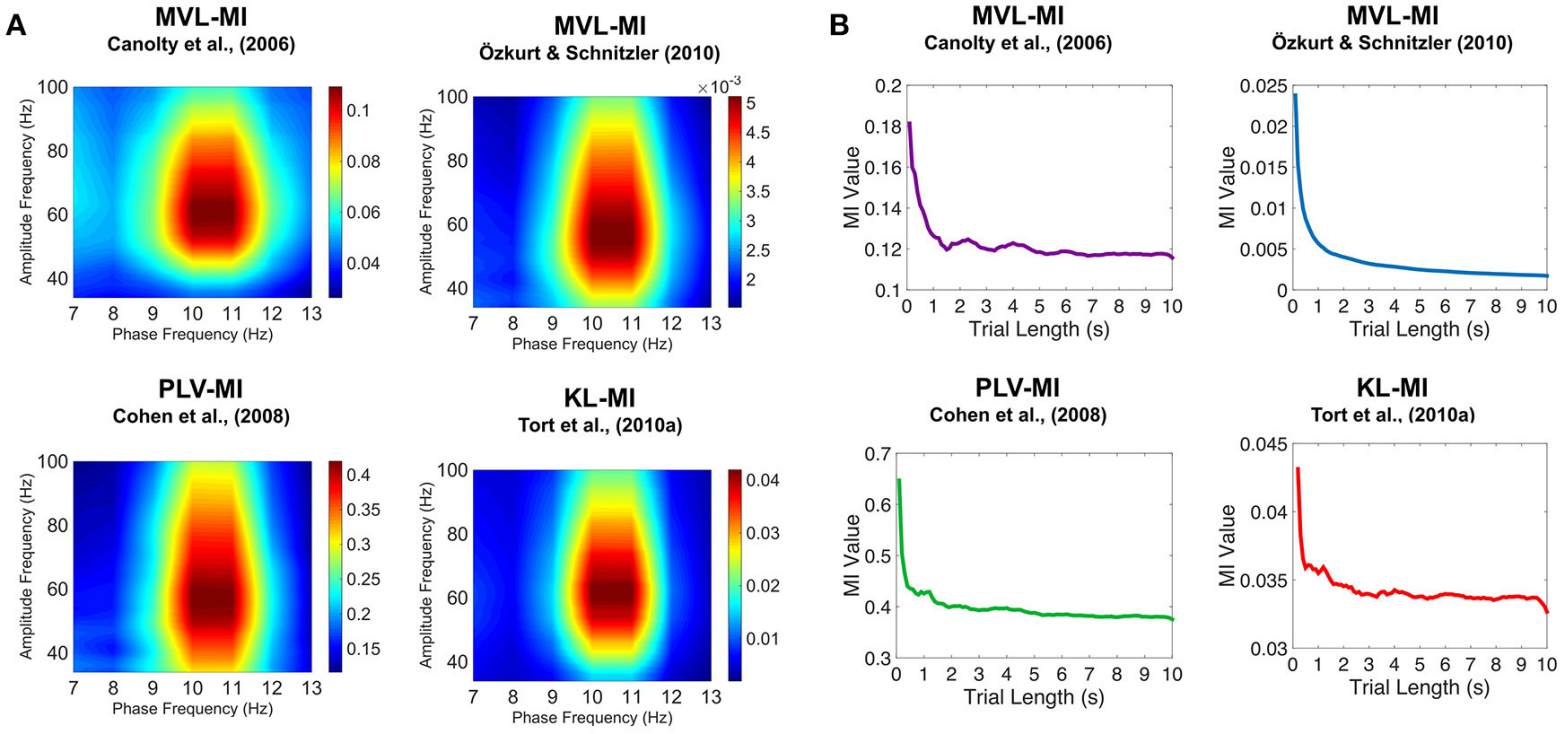
Results of the simulated PAC analysis. **(A)** Phase-amplitude comodulograms produced using the MVL-MI-Canolty, MVL-MI-Özkurt, PLV-MI-Cohen, and KL-MI-Tort algorithms were able to successfully detect the 1.2 s of simulated coupling between 10 Hz phase and 50–70 Hz amplitude. **(B)** The coupling between 10 Hz phase and 60 Hz amplitude was calculated as a function of simulated data trial length. For trial data under 1 s, all four algorithms produced artificially inflated PAC.

To further validate our PAC results, we generated simulated data with known alpha-gamma coupling (10–11 Hz phase, 50–70 Hz amplitude). Using the same MATLAB code as for the MEG data, we were able to successfully detect this alpha-gamma PAC using the MVL-MI-Canolty, MVL-MI-Özkurt, PLV-MI-Cohen and KL-MI-Tort algorithms (Figure 5A). By varying the trial length of the simulated data, we found that PAC values were affected by trial length, with data segments under 1 s producing artificially inflated PAC (Figure 5B).

## Discussion

This article has outlined various steps involved in the detection and validation of PAC in a visual MEG dataset (data shared at: https://doi.org/10.6084/m9.figshare.c.3819106.v1), utilizing the open-source Fieldtrip toolbox (Oostenveld et al., 2010) and customized Matlab scripts (all scripts shared at: https://github.com/neurofractal/sensory_PAC). We first confirmed that presentation of the visual grating was accompanied by decreases in alpha power (8–13 Hz) and increases in gamma power (>40 Hz) within visual area V1. Although this may seem redundant given the wealth of evidence for alpha and gamma oscillations in visual processing (Hoogenboom et al., 2006; Bonnefond and Jensen, 2015; Michalareas et al., 2016), it is crucial to establish clear increases/decreases in the power spectrum at two distinct frequencies as a first step in MEG-PAC analysis (Aru et al., 2015; Hyafil et al., 2015). Using four PAC algorithms, we showed that visual responses obtained from area V1 displayed a general increase in alpha-gamma PAC as expected (Voytek et al., 2010; Spaak et al., 2012; Bonnefond and Jensen, 2015). However, it is important to note that specific patterns of coupling depended on the algorithm selected. The MVL-MI-Canolty algorithm showed large increases in PAC during the grating period, covering almost the entire alpha and gamma frequency ranges, most likely as a result of MI values being biased by increases in high-frequency power following presentation of the visual grating (Canolty et al., 2006). This approach is therefore less suitable for detecting PAC between separate periods of data and/or trials. The MVL-MI-Özkurt algorithm, which normalizes the MI value by high amplitude power, along with the PLV-MI-Cohen algorithm produced a much more constrained pattern of significant alpha-gamma PAC, with peaks between 9–11 Hz phase and 50–70 Hz amplitude. Whilst the KL-MI-Tort approach also showed a general increase in alpha-gamma PAC around 9–11 Hz, none of the phase-amplitude clusters reached significance. This may be due to the relatively short number of trials used in the experiment, the low signal-to-noise ratio of MEG recordings (Goldenholz et al., 2009), variations in the peak alpha and gamma oscillatory frequencies (Muthukumaraswamy et al., 2009), combined with the fact that the KL-MI-Tort approach is relatively conservative (van Driel et al., 2015). More generally, it is important to emphasize that all four PAC metrics are highly sensitive to a range of factors (Dvorak and Fenton, 2014; Aru et al., 2015), which are often hard to control (Berman et al., 2012), resulting in both type I and type II statistical errors.

One such issue is the presence of non-sinusoidal sawtooth-like oscillations in electrophysiological data, which can result in spurious PAC (Lozano-Soldevilla et al., 2016), especially when phase is obtained with wide band-pass filters. By computing the ratio between rise-time and decay-time of alpha oscillations within area V1, we showed that non-sinusoidal oscillations did not differ between baseline and grating periods, and are unlikely to account for our results. Another issue in trial-based PAC analysis is data length, with some previous reports suggesting that 10 s or more is required for detecting theta-gamma coupling (Dvorak and Fenton, 2014; Aru et al., 2015). However, using simulated alpha-gamma PAC we determined that 1 s of data was sufficient to obtain stable estimates. We encourage the reader to run similar follow-up analyses after finding significant PAC to check for spurious coupling caused by, for example, non-sinusoidal oscillations (Jensen et al., 2016; Lozano-Soldevilla et al., 2016) and/or insufficiently long trials (Dvorak and Fenton, 2014).

### Practical considerations for PAC analysis

Cross-frequency coupling is gaining significant interest within the electrophysiological community (Canolty and Knight, 2010; Dvorak and Fenton, 2014; Aru et al., 2015; Hyafil et al., 2015), and therefore it is important for researchers to consider the methodological pitfalls and caveats which commonly arise during PAC analysis. Firstly, due to the presence of edge artifacts at the start and end of time-series created by bandpass filtering, which can result in artefactual PAC (Kramer et al., 2008), sufficient padding should be included around trials. Concatenating data from separate trials to create longer data segments results in similar edge artifacts (Kramer et al., 2008), and should be avoided. Secondly, if the bandwidth of the filter used to extract the amplitude does not contain the side-bands of the modulating phase frequency, PAC cannot be detected even if present (Dvorak and Fenton, 2014). The use of a variable band-pass filter which scales with amplitude frequency, can alleviate this issue and improve the sensitivity of detecting PAC (Berman et al., 2012; Voloh et al., 2015). Thirdly, periods which contain non-stationary periods should be avoided. This includes sensory evoked potentials which induce correlations between frequency bands via phase reset (Sauseng et al., 2007), and can be misinterpreted as PAC (Aru et al., 2015). For this reason, we did not analyse the first 300 ms following visual grating presentation, due to the presence of visual evoked potentials (Di Russo et al., 2002). Fourth, given that PAC algorithms produce values ranging from 0 to 1, data are commonly not normally distributed, and therefore the use of non-parametric statistics is paramount. Whilst surrogate data are often employed (Tort et al., 2010b; Aru et al., 2015), this may not be possible where data are organized into short trials and temporal correlations between surrogate and true time-series are high (Dvorak and Fenton, 2014). Therefore, to assess changes in PAC, using a baseline period or contrasting between conditions, combined with non-parametric statistics may prove to be a useful alternative for sensory neurocognitive research.

## Limitations

This study has compared four PAC algorithms (Canolty et al., 2006; Cohen, 2008; Tort et al., 2010b; Özkurt and Schnitzler, 2011), which are among the most commonly used approaches in sensory EEG/MEG research (Mathewson et al., 2011; Khan et al., 2013; Bonnefond and Jensen, 2015; Cho et al., 2015). However, these only comprise a small subset of the available algorithms designed to quantify PAC (Canolty and Knight, 2010; Hyafil et al., 2015). There have also been advances in measuring transient changes in PAC (Dvorak and Fenton, 2014), directed PAC (Jiang et al., 2015) and algorithms designed for spontaneous neural activity (Florin and Baillet, 2015; Weaver et al., 2016). A more comprehensive evaluation of algorithms and their application to real-world electrophysiological data is beyond the scope of this article, but would nevertheless benefit the field of cross-frequency coupling. Secondly, in order to detect alpha-gamma PAC within visual area V1, we used a broad filter bandwidth, defined as ±0.4 times the amplitude center-frequency. Consequently, the alpha-gamma comodulograms will be unable to differentiate between adjacent gamma sub-bands, which have been proposed to fulfill differing neurocognitive roles (Buzsáki and Wang, 2012; Bosman et al., 2014), and patterns of PAC (Vaz et al., 2017). However, for the visual MEG data presented here, there was only an increase in gamma power within one band (40–70 Hz), and therefore the smearing of adjacent sub-bands is unlikely. Finally, we have focussed on PAC within the visual cortex, which is known to display highly sinusoidal alpha oscillations (Tort et al., 2010a). However, there are many examples of non-sinusoidal brain oscillations caused by physiological neuronal spiking patterns (Fontanini and Katz, 2005), including hippocampal theta (4–8 Hz) and sensorimotor mu (9–11 Hz) rhythms (Lozano-Soldevilla et al., 2016; Scheffer-Teixeira and Tort, 2016), which are indicative of behavior and disease states (Cole and Voytek, 2017). Therefore, whilst non-sinusoidal oscillations generate spurious PAC, this does not mean that these oscillations are uninteresting, but simply that common PAC algorithms, such as, the ones employed in this article, are ill-suited for these scenarios. Where non-sinusoidal oscillations are present, PAC analysis could proceed by correcting for non-uniform phase distributions (e.g., van Driel et al., 2015) in order to disentangle nested oscillations from neural spiking (Vaz et al., 2017).

## Conclusion

In conclusion, we have outlined the key analysis steps for detecting changes in alpha-gamma PAC during sensory processing, using an example visual MEG dataset. While alpha-gamma PAC was shown to increase, the specific patterns of alpha-gamma coupling depended upon the specific algorithm employed. Follow-up analyses showed that these results were not driven by non-sinusoidal oscillations or insufficient data. In future, we hope that a variety of PAC algorithms will be implemented alongside existing open-source MEG toolboxes (Oostenveld et al., 2010; Tadel et al., 2011; Gramfort et al., 2014), with detailed guidance and advice, so that PAC can form a natural analysis step in electrophysiological research.

## Author contributions

RS, KK, and GR co-designed the study and wrote the manuscript. RS collected the data, carried out the analysis, and organized the data and code for sharing.

### Conflict of interest statement

The authors declare that the research was conducted in the absence of any commercial or financial relationships that could be construed as a potential conflict of interest.

## Footnotes

^1^Due to inconsistent naming practices, we refer to the quantitative value of PAC as the modulation index (MI) across all four approaches.

## Supplementary material

MATLAB code for all reported analyses is available in the supplementary materials, along with the indices of rejected trials.
