# Supplementary material for: The Detection of Phase Amplitude Coupling during Sensory Processing
Source: Front Neurosci. 2017 Sep 1;11:487. doi: 10.3389/fnins.2017.00487 (PMC5585190; doi:10.3389/fnins.2017.00487)
Supplement: Supplementary file 1 [file DataSheet1.DOCX]

Supplementary Material

The Detection of Phase Amplitude Coupling During Sensory Processing

Robert A. Seymour*, Gina Rippon, Klaus Kessler

*** Correspondence:** Corresponding Author: seymourr@aston.ac.uk

# Supplementary Analysis Code

The MATLAB code presented here will allow the reader to reproduce all analyses reported in the article, in conjunction with the openly available MEG data. To download this code please visit <https://github.com/neurofractal/sensory_PAC>, and to download the MEG data please visit <https://doi.org/10.6084/m9.figshare.c.3819106.v1>.

Software/hardware dependencies include:

- MATLAB (scripts tested on version 2014b and above)
- Fieldtrip Toolbox v20161024
- Windows PC (scripts may also work on different operating system, but have not been fully tested).
- A permanent copy of the scripts and accompanying functions can be downloaded from <https://doi.org/10.6084/m9.figshare.5297032>
- Updates to the code can be tracked from <https://github.com/neurofractal/sensory_PAC>
  1. **1_preprocessing_elektra_frontiers_PAC.m**

%%

%%%%%%%%%%%%%%%%%%%%%%%%%%%%%%%%%%%%%%%%%%%%%%%%%%%%%%%%%%%%%%%%%%%%%%%%%%%

%

% 1_preprocessing_elekta_frontiers_PAC.m

%

% Matlab script to perform SIMPLE preprocessing using the Fieldtrip

% toolbox (common preprocessing, visualisation and artefact rejection

% steps).

%

% Here we use visual grating data, obtained from the Aston Brain Centre,

% Birmingham (UK), using a Neuromag Elekta (Triux 306 channel) MEG scanner.

% N.B. The trigger code for the onset of the visual grating is 'STI005'.

%

% Output = data_clean_noICA

%

% Written by Robert Seymour - May 2017

%

% Please note that these scripts have been optimised for the Windows

% operating system and MATLAB versions about 2014b.

%

% Runnng time: 15 minutes (requires user to manually inspect trial

% variances and enter the bad trial numbers - see bad_trial_indices.tsv)

%

%%%%%%%%%%%%%%%%%%%%%%%%%%%%%%%%%%%%%%%%%%%%%%%%%%%%%%%%%%%%%%%%%%%%%%%%%%%

%% Load computer-specific information

restoredefaultpath;

sensory_PAC;

addpath(fieldtrip_dir);

addpath(genpath(scripts_dir));

ft_defaults

% If you do not run these lines you will have to manually specify:

% - subject = subject list

% - data_dir = directory which contains the MEG & anatomical information

% - scripts_dir = directory with ALL the scripts

% - fieldtrip_dir = directory containing the Fieldtrip toolbox

for i = 1:length(subject)

%% Prerequisites

% Make a new directory in the scripts folder & cd there

mkdir([scripts_dir '\' subject{i}]);

cd([scripts_dir '\' subject{i}]);

% Specify location of the datafile

rawfile = [data_dir '\' subject{i} '\meg\' subject{i} '_visualgrating-task_quat_tsss.fif'];

% Creates log file

diary(sprintf('log %s.out',subject{i}));

c = datestr(clock); %time and date

disp(sprintf('Running preprocessing script for subject{i} %s',subject{i}))

disp(c)

%% Epoching & Filtering

% Epoch the whole dataset into one continous dataset and apply

% the appropriate filters

cfg = [];

cfg.trialfun = 'ft_trialfun_general';

cfg.headerfile = rawfile;

cfg.datafile = rawfile;

cfg.channel = 'MEG';

cfg.trialdef.triallength = Inf;

cfg.trialdef.ntrials = 1;

cfg = ft_definetrial(cfg);

cfg.continuous = 'yes';

cfg.bpfilter = 'yes';

cfg.bpfreq = [0.5 250];

cfg.channel = 'MEG';

cfg.dftfilter = 'yes';

cfg.dftfreq = [50];

alldata = ft_preprocessing(cfg);

% Deal with 50Hz line noise using a bandstop filter

cfg = [];

cfg.bsfilter = 'yes';

cfg.bsfreq = [49.5 50.5];

alldata = ft_preprocessing(cfg,alldata);

% Deal with 100Hz line noise using a bandstop filter

cfg = [];

cfg.bsfilter = 'yes';

cfg.bsfreq = [99.5 100.5];

alldata = ft_preprocessing(cfg,alldata);

% Epoch your filtered data based on a specific trigger

cfg = [];

cfg.trialfun = 'ft_trialfun_general';

cfg.headerfile = rawfile;

cfg.datafile = rawfile;

cfg.channel = 'MEG';

cfg.trialdef.eventtype = 'STI005';

disp('Trigger Value is STI005');

cfg.trialdef.prestim = 2.0; % pre-stimulus interval

cfg.trialdef.poststim = 2.0; % post-stimulus interval

cfg = ft_definetrial(cfg);

data = ft_redefinetrial(cfg,alldata); %redefines the filtered data

% Detrend and demean each trial

cfg = [];

cfg.demean = 'yes';

cfg.detrend = 'yes';

data = ft_preprocessing(cfg,data);

%% Reject Trials

% Display visual trial summary to reject deviant trials.

% You need to load the mag + grad separately due to different scales.

% Please refer to the .tsv file for indices of rejected trials.

cfg = [];

cfg.method = 'summary';

cfg.keepchannel = 'yes';

cfg.channel = 'MEGMAG';

clean1 = ft_rejectvisual(cfg, data);

% Now load this

cfg.channel = 'MEGGRAD';

clean2 = ft_rejectvisual(cfg, clean1);

data = clean2; clear clean1 clean2

close all

%% Save the clean data

data_clean_noICA = data;

save data_clean_noICA data_clean_noICA

clear data_clean_noICA

close all

%% Go back to scripts directory

cd(scripts_dir);

end

- 1. **2_get_source_power.m**

%%%%%%%%%%%%%%%%%%%%%%%%%%%%%%%%%%%%%%%%%%%%%%%%%%%%%%%%%%%%%%%%%%%%%%%%%%%

%

% 2_get_source_power.m

%

% This script computes source-space power for baseline and grating periods

% in the gamma-band (40-60Hz) and then the alpha-band (8-13Hz), using an

% LCMV beamformer.

%

% For source localisation, a 3D cortical mesh of 4002 vertices per

% hemisphere is used, created using Freesurfer and HCP scripts.

%

% A grandaverage is computed for each frequency band and exported to .nii

% and .gii formats. Please use your favorite MRI visualisation software to

% view these whole-brain % power change maps. Examples include: BrainNet

% Viewer (https://www.nitrc.org/projects/bnv/); MRIcron

% (http://people.cas.sc.edu/rorden/mricron/index.html) and Connectome

% Workbench (http://www.humanconnectome.org/software/connectome-workbench).

%

% Written by Robert Seymour June 2017

%

% Please note that these scripts have been optimised for the Windows

% operating system and MATLAB versions about 2014b.

%

% Running Time: 15-20 mins per frequency band

%

%%%%%%%%%%%%%%%%%%%%%%%%%%%%%%%%%%%%%%%%%%%%%%%%%%%%%%%%%%%%%%%%%%%%%%%%%%%

%% Load computer-specific information

restoredefaultpath

sensory_PAC;

addpath(fieldtrip_dir);

addpath(genpath(scripts_dir));

ft_defaults

% If you do not run these lines you will have to manually specify:

% - subject = subject list

% - data_dir = directory which contains the MEG & anatomical information

% - scripts_dir = directory with ALL the scripts

% - fieldtrip_dir = directory containing the Fieldtrip toolbox

%% Arrays to hold source estimates for each subject

sourcepre_all = []; %baseline

sourcepost_all = []; %grating period

%% Pre-load the Conte69 Brain Template from the HCP

conte69brain = ft_read_headshape({[scripts_dir ...

'\Q1-Q6_R440.L.midthickness.4k_fs_LR.surf.gii'],...

[scripts_dir '\Q1-Q6_R440.R.midthickness.4k_fs_LR.surf.gii']});

%% Start Loop

for i=1:length(subject)

%% Load variables required for source analysis

load([scripts_dir '\' subject{i} '\data_clean_noICA.mat']); % non-ICA'd data

load([data_dir '\' subject{i} '\anat\sens.mat']);

load([data_dir '\' subject{i} '\anat\seg.mat']);

% Convert to consistent units

sens = ft_convert_units(sens,'m');

seg = ft_convert_units(seg,'m');

%% Set the current directory

cd([scripts_dir '\' subject{i}])

%% Set bad channel list - can change to specific channels if necessary

chans_included = {'MEG', '-MEG0322', '-MEG2542','-MEG0111','-MEG0532'};

cfg = [];

cfg.channel = chans_included;

data_clean_noICA = ft_preprocessing(cfg,data_clean_noICA);

%% Load 3D 4k Cortical Mesh for L/R hemisphere & Concatenate

sourcespace = ft_read_headshape({[data_dir '\' subject{i} '\anat\'...

subject{i} '.L.midthickness.4k_fs_LR.surf.gii'],[data_dir...

'\' subject{i} '\anat\' subject{i} '.R.midthickness.4k_fs_LR.surf.gii']});

sourcespace = ft_convert_units(sourcespace,'m');

%% Make sure rank of the data is below 64

% determine numcomponent by doing an eig on the covariance matrix

covar = zeros(numel(data_clean_noICA.label));

for itrial = 1:numel(data_clean_noICA.trial)

currtrial = data_clean_noICA.trial{itrial};

covar = covar + currtrial*currtrial.';

end

[V, D] = eig(covar);

D = sort(diag(D),'descend');

D = D ./ sum(D);

Dcum = cumsum(D);

% number of components accounting for 99% of variance in covar matrix

numcomponent = find(Dcum>.99,1,'first');

% Make sure the rank is below 64

if numcomponent > 65

numcomponent = 64;

end

disp(sprintf('\n Reducing the data to %d components \n',numcomponent));

cfg = [];

cfg.method = 'pca';

cfg.updatesens = 'yes';

cfg.channel = chans_included;

comp = ft_componentanalysis(cfg, data_clean_noICA);

cfg = [];

cfg.updatesens = 'yes';

cfg.component = comp.label(numcomponent:end);

data_clean_noICA = ft_rejectcomponent(cfg, comp);

%% Bandpass Filter

cfg = [];

cfg.channel = chans_included;

cfg.bpfilter = 'yes';

cfg.bpfreq = [40 60]; %band-pass filter in the required range

data_filtered = ft_preprocessing(cfg,data_clean_noICA)

%% Here we redefine trials based on the time-points of interest.

% Make sure the timepoints are of equivalent length

cfg = [];

cfg.toilim = [-1.5 -0.3];

datapre = ft_redefinetrial(cfg, data_filtered);

cfg.toilim = [0.3 1.5];

datapost = ft_redefinetrial(cfg, data_filtered);

% Here we are keeping all parts of the trial for your covariance matrix

cfg = [];

cfg.covariance = 'yes';

cfg.covariancewindow = [-1.5 1.5]

avg = ft_timelockanalysis(cfg,data_filtered);

% Time lock analysis for datapre and datapost period

cfg = [];

cfg.covariance='yes';

cfg.covariancewindow = [-1.5 1.5];

avgpre = ft_timelockanalysis(cfg,datapre);

avgpst = ft_timelockanalysis(cfg,datapost);

%% Setup pre-requisites for source localisation

% Create headmodel

cfg = [];

cfg.method = 'singleshell';

headmodel = ft_prepare_headmodel(cfg, seg);

% Load headshape

headshape = ft_read_headshape([data_dir '\' subject{i} '\meg\' subject{i} '_visualgrating-task_quat_tsss.fif']);

headshape = ft_convert_units(headshape,'m');

%% Create leadfields

cfg=[];

cfg.headmodel=headmodel;

cfg.channel= chans_included;

cfg.grid.pos= sourcespace.pos;

cfg.grid.unit ='m';

cfg.grad=sens;

cfg.grid.inside = [1:1:length(cfg.grid.pos)]; %always inside - check manually

cfg.normalize = 'yes';

sourcemodel_virt=ft_prepare_leadfield(cfg);

% Create Figure to Show Forward Solution

figure; hold on;

ft_plot_headshape(headshape)

ft_plot_mesh(sourcespace,'facecolor','w','edgecolor',[0.5, 0.5, 0.5],'facealpha',0.1);

dataV1 = ft_plot_mesh(sourcemodel_virt.pos(1:8004,:),'vertexcolor','k');

ft_plot_sens(sens, 'style', 'black*')

set(gcf,'color','w'); drawnow;

%% Perform source analysis across the mesh

cfg=[];

cfg.keeptrials = 'no';

cfg.channel= chans_included;

cfg.grad = sens;

cfg.senstype = 'MEG';

cfg.method='lcmv';

cfg.grid = sourcemodel_virt;

cfg.grid.unit ='m';

cfg.headmodel=headmodel;

cfg.lcmv.lamda='5%';

cfg.lcmv.fixedori = 'yes';

cfg.lcmv.keepfilter = 'yes';

cfg.lcmv.projectmom = 'no';

cfg.lcmv.normalize = 'yes';

sourceavg=ft_sourceanalysis(cfg, avg);

% use common filter for subsequent source analysis

cfg.grid.filter=sourceavg.avg.filter; %uses the grid from the whole trial average

%Pre-grating

sourcepreS1 = ft_sourceanalysis(cfg, avgpre);

sourcepreS1.pos = conte69brain.pos; % make sure positions are consistent

sourcepre_all{i} = sourcepreS1;

%Post-grating

sourcepstS1=ft_sourceanalysis(cfg, avgpst);

sourcepstS1.pos = conte69brain.pos; % make sure positions are consistent

sourcepost_all{i} = sourcepstS1;

end

%% Compute Source Grand Average in the Gamma Band

cfg =[];

sourcepost_avg = ft_sourcegrandaverage(cfg,sourcepost_all{:});

sourcepre_avg = ft_sourcegrandaverage(cfg,sourcepre_all{:});

%% Compute Percentage Power Change From Baseline

cfg = [];

cfg.parameter = 'pow';

cfg.operation = '((x1-x2)/x2)*100'; % calculate & change

diff = ft_math(cfg,sourcepost_avg,sourcepre_avg);

%% Plot on the Conte69 Brain (doesn't look very good)

figure;ft_plot_mesh(conte69brain, 'vertexcolor', -diff.pow);colormap(hot);colorbar;

%% Interpolate onto MNI template

mri = ft_read_mri([fieldtrip_dir '\template\anatomy\single_subj_T1.nii']);

cfg = [];

cfg.voxelcoord = 'no';

cfg.parameter = 'pow';

cfg.interpmethod = 'nearest';

diffint = ft_sourceinterpolate(cfg, diff, mri);

%% Export to nifti formt and use your favourite MRI software to visualise

cd(scripts_dir);

cfg = [];

cfg.filetype = 'nifti';

cfg.filename = 'group_visual_gamma_grandavg';

cfg.parameter = 'pow';

ft_sourcewrite(cfg,diffint);

% This corresponds to Figure 3A

%% Export to connectome workbench (specfic to my computer)

%system('D:\Software\workbench\bin_windows64\wb_command -volume-to-surface-mapping D:\scripts\PAC_for_frontiers\group_visual_gamma_grandavg.nii D:\Software\workbench\bin_windows64\Conte69_atlas-v2.LR.32k_fs_LR.wb\32k_ConteAtlas_v2\Conte69.L.midthickness.32k_fs_LR.surf.gii D:\scripts\PAC_for_frontiers\group_visual_gamma_grandavg.nii_LEFT.shape.gii -trilinear')

%system('D:\Software\workbench\bin_windows64\wb_command -volume-to-surface-mapping D:\scripts\PAC_for_frontiers\group_visual_gamma_grandavg.nii D:\Software\workbench\bin_windows64\Conte69_atlas-v2.LR.32k_fs_LR.wb\32k_ConteAtlas_v2\Conte69.R.midthickness.32k_fs_LR.surf.gii D:\scripts\PAC_for_frontiers\group_visual_gamma_grandavg.nii_RIGHT.shape.gii -trilinear')

%%%%%%%%%%%%%%%%%%%%%%%%%%%%%%%%%%%%%%%%%%%%%%%%%%%%%%%%%%%%%%%%%%%%%%%%%%%

%

% Now onto the alpha-band (8-13Hz)

%

%%%%%%%%%%%%%%%%%%%%%%%%%%%%%%%%%%%%%%%%%%%%%%%%%%%%%%%%%%%%%%%%%%%%%%%%%%%

cd(scripts_dir)

clear all ; close all

clc

%% Load computer-specific information

sensory_PAC;

addpath(fieldtrip_dir);

ft_defaults

%% Arrays to hold source estimates for each subject

sourcepre_all = []; %baseline

sourcepost_all = []; %grating

%% Pre-load the Conte69 Brain Template from the HCP

conte69brain = ft_read_headshape({[scripts_dir ...

'\Q1-Q6_R440.L.midthickness.4k_fs_LR.surf.gii'],...

[scripts_dir '\Q1-Q6_R440.R.midthickness.4k_fs_LR.surf.gii']});

%% Start Loop

for i=1:length(subject)

%% Load variables required for source analysis

load([scripts_dir '\' subject{i} '\data_clean_noICA.mat']); % non-ICA'd data

load([data_dir '\' subject{i} '\anat\sens.mat']);

load([data_dir '\' subject{i} '\anat\seg.mat']);

% Convert to consistent units

sens = ft_convert_units(sens,'m');

seg = ft_convert_units(seg,'m');

%% Set the current directory

cd([scripts_dir '\' subject{i}])

%% Set bad channel list - can change to specific channels if necessary

chans_included = {'MEG', '-MEG0322', '-MEG2542','-MEG0111','-MEG0532'};

cfg = [];

cfg.channel = chans_included;

data_clean_noICA = ft_preprocessing(cfg,data_clean_noICA);

%% Load 3D 4k Cortical Mesh for L/R hemisphere & Concatenate

sourcespace = ft_read_headshape({[data_dir '\' subject{i} '\anat\'...

subject{i} '.L.midthickness.4k_fs_LR.surf.gii'],[data_dir...

'\' subject{i} '\anat\' subject{i} '.R.midthickness.4k_fs_LR.surf.gii']});

sourcespace = ft_convert_units(sourcespace,'m');

%% Do your timelock analysis on the data & compute covariance

% determine numcomponent by doing an eig on the covariance matrix

covar = zeros(numel(data_clean_noICA.label));

for itrial = 1:numel(data_clean_noICA.trial)

currtrial = data_clean_noICA.trial{itrial};

covar = covar + currtrial*currtrial.';

end

[V, D] = eig(covar);

D = sort(diag(D),'descend');

D = D ./ sum(D);

Dcum = cumsum(D);

numcomponent = find(Dcum>.99,1,'first'); % number of components accounting for 99% of variance in covar matrix

% Make sure the rank is below 64

if numcomponent > 65

numcomponent = 64;

end

disp(sprintf('\n Reducing the data to %d components \n',numcomponent));

cfg = [];

cfg.method = 'pca';

cfg.updatesens = 'yes';

cfg.channel = chans_included;

comp = ft_componentanalysis(cfg, data_clean_noICA);

cfg = [];

cfg.updatesens = 'yes';

cfg.component = comp.label(numcomponent:end);

data_clean_noICA = ft_rejectcomponent(cfg, comp);

%% BP Filter & Select Gradiometers

cfg = [];

cfg.channel = chans_included;

cfg.bpfilter = 'yes'

cfg.bpfreq = [8 13]; %band-pass filter in the required range

data_filtered = ft_preprocessing(cfg,data_clean_noICA)

%% Here we redefine trials based on the time-points of interest.

% Make sure the timepoints are of equivalent length

cfg = [];

cfg.toilim = [-1.5 -0.3];

datapre = ft_redefinetrial(cfg, data_filtered);

cfg.toilim = [0.3 1.5];

datapost = ft_redefinetrial(cfg, data_filtered);

% Here we are keeping all parts of the trial for your covariance matrix

cfg = [];

cfg.covariance = 'yes';

cfg.covariancewindow = [-1.5 1.5]

avg = ft_timelockanalysis(cfg,data_filtered);

% Time lock analysis for datapre and datapost period

cfg = [];

cfg.covariance='yes';

cfg.covariancewindow = [-1.5 1.5]

avgpre = ft_timelockanalysis(cfg,datapre);

avgpst = ft_timelockanalysis(cfg,datapost);

%% Setup pre-requisites for source localisation

% Create headmodel

cfg = [];

cfg.method = 'singleshell';

headmodel = ft_prepare_headmodel(cfg, seg);

% Load headshape

headshape = ft_read_headshape([data_dir '\' subject{i} '\meg\' subject{i} '_visualgrating-task_quat_tsss.fif']);

headshape = ft_convert_units(headshape,'m');

%% Create leadfields

cfg=[];

cfg.headmodel=headmodel;

cfg.channel= chans_included;

cfg.grid.pos= sourcespace.pos;

cfg.grid.unit ='m';

cfg.grad=sens;

cfg.grid.inside = [1:1:length(cfg.grid.pos)]; %always inside - check manually

cfg.normalize = 'yes';

sourcemodel_virt=ft_prepare_leadfield(cfg);

% Create Figure to Show Forward Solution

figure; hold on;

ft_plot_headshape(headshape)

ft_plot_mesh(sourcespace,'facecolor','w','edgecolor',[0.5, 0.5, 0.5],'facealpha',0.1);

dataV1 = ft_plot_mesh(sourcemodel_virt.pos(1:8004,:),'vertexcolor','k');

ft_plot_sens(sens, 'style', 'black*')

set(gcf,'color','w'); drawnow;

%% Perform source analysis across the mesh

cfg=[];

cfg.keeptrials = 'no';

cfg.channel= chans_included;

cfg.grad = sens;

cfg.senstype = 'MEG';

cfg.method='lcmv';

cfg.grid = sourcemodel_virt;

cfg.grid.unit ='m';

cfg.headmodel=headmodel;

cfg.lcmv.lamda='5%';

cfg.lcmv.fixedori = 'yes';

cfg.lcmv.keepfilter = 'yes';

cfg.lcmv.projectmom = 'no';

cfg.lcmv.normalize = 'yes';

sourceavg=ft_sourceanalysis(cfg, avg);

% use common filter for subsequent source analysis

cfg.grid.filter=sourceavg.avg.filter; %uses the grid from the whole trial average

%Pre-grating

sourcepreS1 = ft_sourceanalysis(cfg, avgpre);

sourcepreS1.pos = conte69brain.pos; % make sure positions are consistent

sourcepre_all{i} = sourcepreS1;

%Post-grating

sourcepstS1=ft_sourceanalysis(cfg, avgpst);

sourcepstS1.pos = conte69brain.pos; % make sure positions are consistent

sourcepost_all{i} = sourcepstS1;

end

%% Compute Source Grand Average

cfg =[];

sourcepost_avg = ft_sourcegrandaverage(cfg,sourcepost_all{:});

sourcepre_avg = ft_sourcegrandaverage(cfg,sourcepre_all{:});

%% Take Post-Grating Power from Baseline Power

cfg = [];

cfg.parameter = 'pow';

cfg.operation = '((x1-x2)/x2)*100';

diff = ft_math(cfg,sourcepost_avg,sourcepre_avg);

%% Plot on the Conte69 Brain (deosn't look very good)

figure;ft_plot_mesh(conte69brain, 'vertexcolor', -diff.pow);colormap(hot);colorbar;

%% Interpolate onto MNI template

mri = ft_read_mri([fieldtrip_dir '\template\anatomy\single_subj_T1.nii']);

cfg = [];

cfg.voxelcoord = 'no';

cfg.parameter = 'pow';

cfg.interpmethod = 'nearest';

diffint = ft_sourceinterpolate(cfg, diff, mri);

%% Export to nifti formt and use your favourite MRI software to visualise

cd(scripts_dir);

cfg = [];

cfg.filetype = 'nifti';

cfg.filename = 'group_visual_alpha_grandavg';

cfg.parameter = 'pow';

ft_sourcewrite(cfg,diffint);

% This corresponds to Figure 3B

%% Export to connectome workbench (specfic to my computer)

%system('D:\Software\workbench\bin_windows64\wb_command -volume-to-surface-mapping D:\scripts\PAC_for_frontiers\group_visual_alpha_grandavg.nii D:\Software\workbench\bin_windows64\Conte69_atlas-v2.LR.32k_fs_LR.wb\32k_ConteAtlas_v2\Conte69.L.midthickness.32k_fs_LR.surf.gii D:\scripts\PAC_for_frontiers\group_visual_alpha_grandavg.nii_LEFT.shape.gii -trilinear')

%system('D:\Software\workbench\bin_windows64\wb_command -volume-to-surface-mapping D:\scripts\PAC_for_frontiers\group_visual_alpha_grandavg.nii D:\Software\workbench\bin_windows64\Conte69_atlas-v2.LR.32k_fs_LR.wb\32k_ConteAtlas_v2\Conte69.R.midthickness.32k_fs_LR.surf.gii D:\scripts\PAC_for_frontiers\group_visual_alpha_grandavg.nii_RIGHT.shape.gii -trilinear')

**1.3 3_get_VE_frontiers_PAC.m**

%%

%%%%%%%%%%%%%%%%%%%%%%%%%%%%%%%%%%%%%%%%%%%%%%%%%%%%%%%%%%%%%%%%%%%%%%%%%%%

%

% This script computes a virtual electrode time-series from area V1. Source

% analysis is performed across all vertices of the 3D cortical mesh.

% Vertex locations within visual area V1 are then defined using the

% HCP-MMP 1.0 atlas.

%

% The spatial filters from these vertices are concatenated, multiplied by

% the sensor-level covariance mstrix and a PCA is performed to extract a

% single V1 filter. This is multipled by the sensor-level trial data to

% generate VE_V1.mat.

%

% Please note: It is also perfectly viable to use a volumetric atlas (e.g.

% AAL) to generate this V1 virtual electrode.

%

% Output: VE_V1.mat. This is saved in the sensory_PAC/sub-XX/ directory

%

% Written by Robert Seymour - June 2017

%

% Please note that these scripts have been optimised for the Windows

% operating system and MATLAB versions about 2014b.

%

% Running-time: 15-20 minutes

%

%%%%%%%%%%%%%%%%%%%%%%%%%%%%%%%%%%%%%%%%%%%%%%%%%%%%%%%%%%%%%%%%%%%%%%%%%%%

%% Load computer-specific information

restoredefaultpath

sensory_PAC;

addpath(fieldtrip_dir);

ft_defaults

% If you do not run these lines you will have to manually specify:

% - subject = subject list

% - data_dir = directory which contains the MEG & anatomical information

% - scripts_dir = directory with ALL the scripts

% - fieldtrip_dir = directory containing the Fieldtrip toolbox

%% Preload the HCP atlas

% Here we are using the 4k HCP atlas mesh to define visual ROIs

% from the subject-specific 4k cortical mesh

load([scripts_dir '\' 'atlas_MSMAll_4k.mat']);

atlas = ft_convert_units(atlas,'m');

%% Start Loop

for i=1:length(subject)

%% Load variables required for source analysis

load([scripts_dir '\' subject{i} '\data_clean_noICA.mat']);

load([data_dir '\' subject{i} '\anat\sens.mat']);

load([data_dir '\' subject{i} '\anat\seg.mat']);

sens = ft_convert_units(sens,'m');

seg = ft_convert_units(seg,'m');

%% Set the current directory

cd([scripts_dir '\' subject{i}])

%% Set bad channel list - can change to specific channels if necessary

chans_included = {'MEG', '-MEG0322', '-MEG2542','-MEG0111','-MEG0532'};

cfg = [];

cfg.channel = chans_included;

data_clean_noICA = ft_preprocessing(cfg,data_clean_noICA);

%% Load 3D 4k Cortical Mesh for L/R hemisphere & Concatenate

sourcespace = ft_read_headshape({[data_dir '\' subject{i} '\anat\'...

subject{i} '.L.midthickness.4k_fs_LR.surf.gii'],[data_dir...

'\' subject{i} '\anat\' subject{i} '.R.midthickness.4k_fs_LR.surf.gii']});

sourcespace = ft_convert_units(sourcespace,'m');

%% Do your timelock analysis on the data & compute covariance

% determine numcomponent by doing an eig on the covariance matrix

covar = zeros(numel(data_clean_noICA.label));

for itrial = 1:numel(data_clean_noICA.trial)

currtrial = data_clean_noICA.trial{itrial};

covar = covar + currtrial*currtrial.';

end

[V, D] = eig(covar);

D = sort(diag(D),'descend');

D = D ./ sum(D);

Dcum = cumsum(D);

numcomponent = find(Dcum>.99,1,'first'); % number of components accounting for 99% of variance in covar matrix

% Make sure the rank is below 64

if numcomponent > 65

numcomponent = 64;

end

disp(sprintf('\n Reducing the data to %d components \n',numcomponent));

cfg = [];

cfg.method = 'pca';

cfg.updatesens = 'yes';

cfg.channel = chans_included;

comp = ft_componentanalysis(cfg, data_clean_noICA);

cfg = [];

cfg.updatesens = 'yes';

cfg.component = comp.label(numcomponent:end);

data_clean_noICA = ft_rejectcomponent(cfg, comp);

%% Time-Lock Analysis

cfg = [];

cfg.channel = chans_included;

cfg.covariance = 'yes'; % compute the covariance for single trials, then average

cfg.covariancewindow = [-1.5 1.5]; % compute the covariance for single trials, then average

cfg.preproc.baselinewindow = [-inf 0]; % reapply the baseline correction

cfg.keeptrials = 'no';

timelock1 = ft_timelockanalysis(cfg, data_clean_noICA);

%% Setup pre-requisites for source localisation

% Create headmodel

cfg = [];

cfg.method = 'singleshell';

headmodel = ft_prepare_headmodel(cfg, seg);

% Load headshape

headshape = ft_read_headshape([data_dir '\' subject{i} '\meg\' subject{i} '_visualgrating-task_quat_tsss.fif']);

headshape = ft_convert_units(headshape,'m');

%% Create leadfields

cfg=[];

cfg.headmodel=headmodel;

cfg.channel= chans_included;

cfg.grid.pos= sourcespace.pos;

cfg.grid.unit ='m';

cfg.grad=sens;

cfg.grid.inside = [1:1:length(cfg.grid.pos)]; %always inside - check manually

cfg.normalize = 'yes';

sourcemodel_virt=ft_prepare_leadfield(cfg);

% Create Figure to Show Forward Solution

figure; hold on;

ft_plot_headshape(headshape)

ft_plot_mesh(sourcespace,'facecolor','w','edgecolor',[0.5, 0.5, 0.5],'facealpha',0.1);

dataV1 = ft_plot_mesh(sourcemodel_virt.pos(1:8004,:),'vertexcolor','k');

ft_plot_sens(sens, 'style', 'black*')

set(gcf,'color','w'); drawnow;

%% Perform source analysis across the mesh

cfg=[];

cfg.keeptrials = 'no';

cfg.channel= chans_included;

cfg.grad = sens;

cfg.senstype = 'MEG';

cfg.method='lcmv';

cfg.grid = sourcemodel_virt;

cfg.grid.unit ='m';

cfg.headmodel=headmodel;

cfg.lcmv.lamda='5%';

cfg.lcmv.fixedori = 'yes';

cfg.lcmv.keepfilter = 'yes';

cfg.lcmv.projectmom = 'no';

cfg.lcmv.normalize = 'yes';

source=ft_sourceanalysis(cfg, timelock1);

%% Compute the V1 virtual electrode

% Get spatial filters from 182 V1 vertices (left and right hemisphere)

indx_V1_L = find(ismember(atlas.parcellationlabel,'L_V1_ROI')); % find index of the required label

sel = find(atlas.parcellation==indx_V1_L);

vertices_V1_L = cat(1,source.avg.filter{sel});

vertices_V1_L = vertices_V1_L(12:end,:);

indx_V1_R = find(ismember(atlas.parcellationlabel,'R_V1_ROI')); % find index of the required label

sel = find(atlas.parcellation==indx_V1_R);

vertices_V1_R = cat(1,source.avg.filter{sel});

vertices_V1_R = vertices_V1_R(12:end,:);

% Perform PCA on concatenated filters * sensor-level covar matrix

F = vertcat(vertices_V1_L,vertices_V1_R);

[u,s,v] = svd(F*timelock1.cov*F');

filter = u'*F;

% Create VE using this filter

VE_V1 = [];

VE_V1.label = {'VE_V1'};

VE_V1.trialinfo = data_clean_noICA.trialinfo;

for sub=1:(length(data_clean_noICA.trialinfo))

% note that this is the non-filtered "raw" data

VE_V1.time{sub} = data_clean_noICA.time{sub};

VE_V1.trial{sub}(1,:) = filter(1,:)*data_clean_noICA.trial{sub}(:,:);

end

% Preserve .sampleinfo field to avoid warnings later

VE_V1.sampleinfo = data_clean_noICA.sampleinfo;

% Save

save VE_V1 VE_V1

%% Create TFR of the VE

% Note - these results are not shown in the manuscript

cfg = [];

cfg.method = 'mtmconvol';

cfg.output = 'pow';

cfg.pad = 'nextpow2'

cfg.foi = 20:1:100;

cfg.toi = -2.0:0.02:2.0;

cfg.t_ftimwin = ones(length(cfg.foi),1).*0.5;

cfg.tapsmofrq = ones(length(cfg.foi),1).*8;

multitaper = ft_freqanalysis(cfg, VE_V1);

%% Plot

cfg = [];

cfg.ylim = [40 100];

cfg.baseline = [-1.5 0];

cfg.xlim = [-0.5 1.5];

figure; ft_singleplotTFR(cfg, multitaper);

title(sprintf('%s',subject{i}));

colormap(jet)

end

**1.4 4_calc_pow_change.m**

%%

%%%%%%%%%%%%%%%%%%%%%%%%%%%%%%%%%%%%%%%%%%%%%%%%%%%%%%%%%%%%%%%%%%%%%%%%%%%

%

% 4_calc_pow_change.m

%

% This script computes the percentage change in oscillatory power

% between baseline and grating periods, from 1-100Hz using data from the

% V1 virtual electrode. A multi-taper approach is employed with a 0.5s

% window and +-8Hz frequency smoothing.

%

% A figure is then produced to show each subject's percentage power change

% as well as the average power change response.

%

% Written by: Robert Seymour, June 2017

%

% Please note that these scripts have been optimised for the Windows

% operating system and MATLAB versions about 2014b.

%

% Runtime: 10-15 minutes

%

%%%%%%%%%%%%%%%%%%%%%%%%%%%%%%%%%%%%%%%%%%%%%%%%%%%%%%%%%%%%%%%%%%%%%%%%%%%

%% Load computer-specific information

restoredefaultpath

sensory_PAC;

addpath(fieldtrip_dir);

addpath(genpath(scripts_dir));

ft_defaults

% If you do not run these lines you will have to manually specify:

% - subject = subject list

% - data_dir = directory which contains the MEG & anatomical information

% - scripts_dir = directory with ALL the scripts

% - fieldtrip_dir = directory containing the Fieldtrip toolbox

perc_change_all = []; % variabe to hold output from all subjects

%% Start loop for each subject

for i = 1:length(subject)

% cd to the right place and load the V1 virtual electrode

load([scripts_dir '\' subject{i} '\VE_V1.mat']);

load([scripts_dir '\' subject{i} '\data_clean_noICA.mat']);

VE_V1.sampleinfo = data_clean_noICA.sampleinfo;

% Calculate Power in Grating & Baseline Periods

cfg = [];

cfg.method = 'mtmconvol';

cfg.output = 'pow';

cfg.pad = 'nextpow2';

cfg.foi = 1:1:100; %1-100Hz

cfg.toi = 0.3:0.02:1.5; %300-1200ms period

cfg.t_ftimwin = ones(length(cfg.foi),1).*0.5;

cfg.tapsmofrq = ones(length(cfg.foi),1).*8;

multitaper_post = ft_freqanalysis(cfg, VE_V1);

cfg.toi = -1.5:0.02:-0.3; %1200ms baseline period

multitaper_pre = ft_freqanalysis(cfg, VE_V1);

% Calculate % change by averaging over time

perc_change = (squeeze(mean(multitaper_post.powspctrm,3))...

- squeeze(mean(multitaper_pre.powspctrm,3)));

perc_change(:,:) = perc_change(:,:)./squeeze(mean(multitaper_pre.powspctrm,3));

perc_change(:,:) = perc_change(:,:)*100;

% Add to array outside the loop

perc_change_all(i,:) = perc_change;

end

%% Create Figure

% This corresponds to Figure 3D

average_change = mean(perc_change_all);

figHandle = figure;

% Add the line to the figure

hold on;

for sub = 1:length(subject)

plot([1:1:100],perc_change_all(sub,:),'LineWidth',3);

hold on;

end

plot([1:1:100],average_change,'k','LineWidth',6);

ylabel('% Power Change');

xlabel('Frequency (Hz)');

set(gca,'FontName','Arial');

set(gca,'FontSize',30);

set(gcf, 'Color', 'w');

**1.5 5_visual_PAC_four_methods.m**

%%

%%%%%%%%%%%%%%%%%%%%%%%%%%%%%%%%%%%%%%%%%%%%%%%%%%%%%%%%%%%%%%%%%%%%%%%%%%%

%

% 5_visual_PAC_four_methods.m

%

% Script to compute PAC comodulograms for the pre and post grating period,

% using visual area V1 data. Four PAC algorithms are currently implemented:

% KL-MI-Tort, MVL-MI-Canolty, MVL-MI-Ozkurt and PLV-MI-Cohen.

%

% Each approach calculates PAC for 64 trials * 16 participants * 7 phase

% frequencies and 34 amplitude frequencies. This will take some time. If

% performance is slow the user might wish to run the algorithms in parallel

% using 4 separate MATLAB windows.

%

% Once completed, the PAC comodulograms are statistically compared

% and the results are plotted. It is worth doing this part separately for

% each algorithm.

%

% Written by Robert Seymour - June 2017

%

% Please note that these scripts have been optimised for the Windows

% operating system and MATLAB versions about 2014b.

%

% Computation time: 4-6 hours

%

%%%%%%%%%%%%%%%%%%%%%%%%%%%%%%%%%%%%%%%%%%%%%%%%%%%%%%%%%%%%%%%%%%%%%%%%%%%

%% Load computer-specific information

restoredefaultpath

sensory_PAC;

addpath(fieldtrip_dir);

addpath(genpath(scripts_dir));

ft_defaults

% If you do not run these lines you will have to manually specify:

% - subject = subject list

% - data_dir = directory which contains the MEG & anatomical information

% - scripts_dir = directory with ALL the scripts

% - fieldtrip_dir = directory containing the Fieldtrip toolbox

%% Start loop for all subjects

for sub = 1:length(subject)

% Load in data and cd to the right place

cd([scripts_dir '\' subject{sub}])

load([scripts_dir '\' subject{sub} '\VE_V1.mat']);

load([scripts_dir '\' subject{sub} '\data_clean_noICA.mat']);

VE_V1.sampleinfo = data_clean_noICA.sampleinfo;

%%%%%%%%%%%%%%%%%%%%%%%%%%%%%%%%%%%%%%%%%%%%%%%%%%%%%%%%%%%%%%%%%%%%%%%

% Tort et al., (2010)

%%%%%%%%%%%%%%%%%%%%%%%%%%%%%%%%%%%%%%%%%%%%%%%%%%%%%%%%%%%%%%%%%%%%%%%

% Get comod for post grating (0.3 to 1.5s) period

[matrix_post,matrix_post_surrogates] = calc_MI(VE_V1,[0.3 1.5],[7 13],[34 100],'no','yes','tort');

save matrix_post_tort matrix_post;

save matrix_post_tort_surrogates matrix_post_surrogates;

% Get comod for pre grating (-1.5 to -0.3s) period

[matrix_pre,matrix_pre_surrogates] = calc_MI(VE_V1,[-1.5 -0.3],[7 13],[34 100],'no','yes','tort')

save matrix_pre_tort matrix_pre;

save matrix_pre_tort_surrogates matrix_pre_surrogates;

clear matrix_post matrix_pre matrix_post_surrogates matrix_pre_surrogates

%

%%%%%%%%%%%%%%%%%%%%%%%%%%%%%%%%%%%%%%%%%%%%%%%%%%%%%%%%%%%%%%%%%%%%%%

% Ozkurt et al., (2010)

%%%%%%%%%%%%%%%%%%%%%%%%%%%%%%%%%%%%%%%%%%%%%%%%%%%%%%%%%%%%%%%%%%%%%%

% Get comod for post grating (0.3 to 1.5s) period

[matrix_post,matrix_post_surrogates] = calc_MI(VE_V1,[0.3 1.5],[7 13],[34 100],'no','yes','ozkurt');

save matrix_post_ozkurt matrix_post;

save matrix_post_ozkurt_surrogates matrix_post_surrogates;

% Get comod for pre grating (-1.5 to -0.3s) period

[matrix_pre,matrix_pre_surrogates] = calc_MI(VE_V1,[-1.5 -0.3],[7 13],[34 100],'no','yes','ozkurt')

save matrix_pre_ozkurt matrix_pre;

save matrix_pre_ozkurt_surrogates matrix_pre_surrogates;

clear matrix_post matrix_pre matrix_post_surrogates matrix_pre_surrogates

%%%%%%%%%%%%%%%%%%%%%%%%%%%%%%%%%%%%%%%%%%%%%%%%%%%%%%%%%%%%%%%%%%%%%%%

% Canolty et al., (2006)

%%%%%%%%%%%%%%%%%%%%%%%%%%%%%%%%%%%%%%%%%%%%%%%%%%%%%%%%%%%%%%%%%%%%%%%

% Get comod for post grating (0.3 to 1.5s) period

[matrix_post,matrix_post_surrogates] = calc_MI(VE_V1,[0.3 1.5],[7 13],[34 100],'no','yes','canolty');

save matrix_post_canolty matrix_post;

save matrix_post_canolty_surrogates matrix_post_surrogates;

% Get comod for pre grating (-1.5 to -0.3s) period

[matrix_pre,matrix_pre_surrogates] = calc_MI(VE_V1,[-1.5 -0.3],[7 13],[34 100],'no','yes','canolty')

save matrix_pre_canolty matrix_pre;

save matrix_pre_canolty_surrogates matrix_pre_surrogates;

clear matrix_post matrix_pre matrix_post_surrogates matrix_pre_surrogates

%%%%%%%%%%%%%%%%%%%%%%%%%%%%%%%%%%%%%%%%%%%%%%%%%%%%%%%%%%%%%%%%%%%%%%%

% PLV

%%%%%%%%%%%%%%%%%%%%%%%%%%%%%%%%%%%%%%%%%%%%%%%%%%%%%%%%%%%%%%%%%%%%%%%

% Get comod for post grating (0.3 to 1.5s) period

[matrix_post,matrix_post_surrogates] = calc_MI(VE_V1,[0.3 1.5],[7 13],[34 100],'no','yes','PLV');

save matrix_post_PLV matrix_post;

save matrix_post_PLV_surrogates matrix_post_surrogates;

% Get comod for pre grating (-1.5 to -0.3s) period

[matrix_pre,matrix_pre_surrogates] = calc_MI(VE_V1,[-1.5 -0.3],[7 13],[34 100],'no','yes','PLV')

save matrix_pre_PLV matrix_pre;

save matrix_pre_PLV_surrogates matrix_pre_surrogates;

clear matrix_post matrix_pre matrix_post_surrogates matrix_pre_surrogates

end

%%

%%%%%%%%%%%%%%%%%%%%%%%%%%%%%%%%%%%%%%%%%%%%%%%%%%%%%%%%%%%%%%%%%%%%%%%

% Statistical Analysis

%%%%%%%%%%%%%%%%%%%%%%%%%%%%%%%%%%%%%%%%%%%%%%%%%%%%%%%%%%%%%%%%%%%%%%%

%

%%%%%%%%%%%%%%%%%%%%%%%%%%%%%%%%%%%%%%%%%%%%%%%%%%%%%%%%%%%%%%%%%%%%%%%

% Canolty et al., (2006) - MVL

%%%%%%%%%%%%%%%%%%%%%%%%%%%%%%%%%%%%%%%%%%%%%%%%%%%%%%%%%%%%%%%%%%%%%%%

[stat_canolty] = get_PAC_stats('matrix_post_canolty.mat',...

'matrix_pre_canolty',[7 13],[34 100],subject,scripts_dir,0)

[stat_canolty_surr] = get_PAC_stats('matrix_post_canolty_surrogates.mat',...

'matrix_pre_canolty_surrogates',[7 13],[34 100],subject,scripts_dir,1)

make_smoothed_comodulograms(stat_canolty, [7 13], [34 100]);

title('Canolty 2006 - no surr');

make_smoothed_comodulograms(stat_canolty_surr, [7 13], [34 100]);

title('Canolty 2006 - with surr');

%

%%%%%%%%%%%%%%%%%%%%%%%%%%%%%%%%%%%%%%%%%%%%%%%%%%%%%%%%%%%%%%%%%%%%%%%

% Ozkurt et al., (2011) - MVL

%%%%%%%%%%%%%%%%%%%%%%%%%%%%%%%%%%%%%%%%%%%%%%%%%%%%%%%%%%%%%%%%%%%%%%%

[stat_ozkurt] = get_PAC_stats('matrix_post_ozkurt.mat',...

'matrix_pre_ozkurt',[7 13],[34 100],subject,scripts_dir,0)

[stat_ozkurt_surr] = get_PAC_stats('matrix_post_ozkurt_surrogates.mat',...

'matrix_pre_ozkurt_surrogates',[7 13],[34 100],subject,scripts_dir,1)

make_smoothed_comodulograms(stat_ozkurt, [7 13], [34 100]);

title('Okzurt 2011 - no surr');

make_smoothed_comodulograms(stat_ozkurt_surr, [7 13], [34 100]);

title('Okzurt 2011 - with surr');

%

%%%%%%%%%%%%%%%%%%%%%%%%%%%%%%%%%%%%%%%%%%%%%%%%%%%%%%%%%%%%%%%%%%%%%%%

% Cohen et al., (2008) - PLV

%%%%%%%%%%%%%%%%%%%%%%%%%%%%%%%%%%%%%%%%%%%%%%%%%%%%%%%%%%%%%%%%%%%%%%%

[stat_PLV] = get_PAC_stats('matrix_post_PLV.mat',...

'matrix_pre_PLV',[7 13],[34 100],subject,scripts_dir,0)

[stat_PLV_surr] = get_PAC_stats('matrix_post_PLV_surrogates.mat',...

'matrix_pre_PLV_surrogates',[7 13],[34 100],subject,scripts_dir,1)

make_smoothed_comodulograms(stat_PLV, [7 13], [34 100]);

title('Cohen PLV - no surr');

make_smoothed_comodulograms(stat_PLV_surr, [7 13], [34 100]);

title('Cohen PLV - with surr');

%

%%%%%%%%%%%%%%%%%%%%%%%%%%%%%%%%%%%%%%%%%%%%%%%%%%%%%%%%%%%%%%%%%%%%%%%

% Tort et al., (2010) - MI

%%%%%%%%%%%%%%%%%%%%%%%%%%%%%%%%%%%%%%%%%%%%%%%%%%%%%%%%%%%%%%%%%%%%%%%

[stat_tort] = get_PAC_stats('matrix_post_tort.mat',...

'matrix_pre_tort.mat',[7 13],[34 100],subject,scripts_dir,0)

[stat_tort_surr] = get_PAC_stats('matrix_post_tort_surrogates.mat',...

'matrix_pre_tort_surrogates.mat',[7 13],[34 100],subject,scripts_dir,1)

make_smoothed_comodulograms(stat_tort, [7 13], [34 100]);

title('Tort 2010 - no surr');

make_smoothed_comodulograms(stat_tort_surr, [7 13], [34 100]);

title('Tort 2010 - with surr');

**1.6 6_check_non_sinusoidal.m**

%%

%%%%%%%%%%%%%%%%%%%%%%%%%%%%%%%%%%%%%%%%%%%%%%%%%%%%%%%%%%%%%%%%%%%%%%%%%%%

%

% 6_check_non_sinusoidal.m

%

% Script to quantify the non-sinusoidal properties of oscillations within

% vidual area V1. Data is concatenated and the rise-time versus decay time

% of low frequency alpha oscillations is calculated. This ratio is

% compared between baseline and grating periods.

%

% Written by Robert Seymour, June 2017.

%

% Please note that these scripts have been optimised for the Windows

% operating system and MATLAB versions about 2014b.

%

%%%%%%%%%%%%%%%%%%%%%%%%%%%%%%%%%%%%%%%%%%%%%%%%%%%%%%%%%%%%%%%%%%%%%%%%%%%

%% Load computer-specific information

restoredefaultpath

sensory_PAC;

addpath(fieldtrip_dir);

addpath(genpath(scripts_dir));

ft_defaults

% If you do not run these lines you will have to manually specify:

% - subject = subject list

% - data_dir = directory which contains the MEG & anatomical information

% - scripts_dir = directory with ALL the scripts

% - fieldtrip_dir = directory containing the Fieldtrip toolbox

%% Concatenate all VE data into single variable

VE_V1_concat = [];

% Start loop for all subjects

for sub = 1:length(subject)

% Load in data

load([scripts_dir '\' subject{sub} '\VE_V1.mat']);

% Append

if sub == 1

VE_V1_concat = VE_V1;

else

cfg = [];

VE_V1_concat = ft_appenddata(cfg,VE_V1_concat,VE_V1);

end

end

%% Calculate the Rise Time: Decay Time for Gratig & Baseline Periods

stats_all = []; % Variable to hold the output from the t-test

p_all = []; % Variable to hold the p-value from the t-test

count = 1; % For use within the loop

figure; % Create figure

% N.B. Please ignore the warning: reconstructing sampleinfo by assuming

% that the trials are consecutive segments of a continuous recording. The

% warning is raised because the concatenated data do not have a

% .sampleinfo field. It does not affect the analysis.

% Start loop for phases 7-13Hz

for phase = 7:13

% Use check_non_sinusoidal_rise_decay function for grating and baseline

% periods

[ratios_post_grating,time_to_decay_all,time_to_peak_all] = ...

check_non_sinusoidal_rise_decay(VE_V1_concat,[0.3 1.5],phase);

[ratios_pre_grating,time_to_decay_all,time_to_peak_all] = ...

check_non_sinusoidal_rise_decay(VE_V1_concat,[-1.5 -0.3],phase);

% Create two overalapping histograms and add to subplot

subplot(2,4,count); histogram(ratios_post_grating); hold on;

histogram(ratios_pre_grating);

xlabel('Time to Peak:Decay'); ylabel('Count');

legend({'Ratio Pre-Grating' 'Ratio Post-Grating'});

% Do a t-test to check for difference between ratio values pre &

% post-grating

[h,p,ci,stats] = ttest(ratios_post_grating,ratios_pre_grating);

title([num2str(phase) 'Hz ; p = ' num2str(p)]);

% Add this to the varibles outsode the loop for all phases

stats_all{count} = stats;

p_all(count) = p;

count = count+1;

disp(['Phase ' num2str(phase)]);

end

**1.7 7_simulated_PAC_analysis.m**

%%%%%%%%%%%%%%%%%%%%%%%%%%%%%%%%%%%%%%%%%%%%%%%%%%%%%%%%%%%%%%%%%%%%%%%%%%%

%

% 7_simulated_PAC_analysis.m

%

% This script produces synthesised PAC between 10-11Hz and 50-70Hz. 4 PAC

% algorithms are then applied to determine how well they recover this

% coupling.

%

% PAC between 10Hz and 60Hz is then calculated as a function of

% data length, to determine how many seconds of data are needed for

% reliable estimates.

%

% N.B. Due to the use of random noise values, the resulting plots may vary

% slightly from the Seymour, Kessler & Rippon (2017) manuscript.

%

% Written by: Robert Seymour, June 2017

%

% Please note that these scripts have been optimised for the Windows

% operating systm and MATLAB versions about 2014b.

%

% Runtime: 10 minutes

%

%%%%%%%%%%%%%%%%%%%%%%%%%%%%%%%%%%%%%%%%%%%%%%%%%%%%%%%%%%%%%%%%%%%%%%%%%%%

%% Load computer-specific information

restoredefaultpath

sensory_PAC;

addpath(fieldtrip_dir);

addpath(genpath(scripts_dir));

ft_defaults

% If you do not run these lines you will have to manually specify:

% - data_dir = directory which contains the MEG & anatomical information

% - scripts_dir = directory with ALL the scripts

% - fieldtrip_dir = directory containing the Fieldtrip toolbox

%% Show an example of the Synthesised PAC

[s_final, snr] = synthesize_pac(2);

figure; plot(s_final(1:1000));

%% Create 64 different SNRs

rng('default'); rng(1)

snr_array = rand(64,1)*3

%% Create Fieldtrip-like Virtual Electrode with 64 trials of synthesised PAC

VE_PAC = [];

VE_PAC.label = {'PAC'};

for i = 1:64 % for every trial

% syntheise PAC using a variable noise value

[s_final, snr] = synthesize_pac(snr_array(i));

VE_PAC.trial{1,i} = s_final(1:10000); % Put simulated PAC into Fieldtrip VE

VE_PAC.time{1,i} = 0.001:0.001:10; % Create 10s worth of PAC

VE_PAC.trialinfo(i,1) = 1;

disp(['Trial ' num2str(i)]);

VE_PAC.sampleinfo(i,:) = [10000*i 10000*i+9999];

end

%% Create comodulogram using the Ozkurt method

canolty_PAC = calc_MI(VE_PAC,[0.3 1.5],[7 13],[34 100],'no','no','canolty');

figure; xticks = [7:1:13];

pcolor([7:1:13],[34:2:100],canolty_PAC); shading(gca,'interp');

colormap(jet);

set(gca,'FontSize',30);

xlabel('Phase Frequency (Hz)','FontSize',25);ylabel('Amplitude Frequency (Hz)','FontSize',25);

%title('MVL-MI-Canolty');

set(gca,'FontName','Arial');

set(gca,'XTick',xticks);

%% Create comodulogram using the Ozkurt method

ozkurt_PAC = calc_MI(VE_PAC,[0.3 1.5],[7 13],[34 100],'no','no','ozkurt');

figure; xticks = [7:1:13];

pcolor([7:1:13],[34:2:100],ozkurt_PAC); shading(gca,'interp');

colormap(jet); colorbar;

set(gca,'FontSize',30);

xlabel('Phase Frequency (Hz)','FontSize',25);ylabel('Amplitude Frequency (Hz)','FontSize',25);

%title('MVL-MI-ozkurt');

set(gca,'FontName','Arial');

set(gca,'XTick',xticks);

%% Create comodulogram using the Tort method

tort_PAC = calc_MI(VE_PAC,[0.3 1.5],[7 13],[34 100],'no','no','tort');

figure; xticks = [7:1:13];

pcolor([7:1:13],[34:2:100],tort_PAC); shading(gca,'interp');

colormap(jet); colorbar;

set(gca,'FontSize',30);

xlabel('Phase Frequency (Hz)','FontSize',25);ylabel('Amplitude Frequency (Hz)','FontSize',25);

%title('MVL-MI-Tort');

set(gca,'FontName','Arial');

set(gca,'XTick',xticks);

%% Create comodulogram using the Cohen PLV method

PLV_PAC = calc_MI(VE_PAC,[0.3 1.5],[7 13],[34 100],'no','no','PLV');

figure; xticks = [7:1:13];

pcolor([7:1:13],[34:2:100],PLV_PAC); shading(gca,'interp');

colormap(jet); colorbar;

set(gca,'FontSize',30);

xlabel('Phase Frequency (Hz)','FontSize',25);ylabel('Amplitude Frequency (Hz)','FontSize',25);

%title('MVL-MI-PLV');

set(gca,'FontName','Arial');

set(gca,'XTick',xticks);

%% How does PAC vary with trial length?

MI_canolty = []; % 0.1-10s in 0.1s steps

for k = 1:100

MI_canolty(k) = calc_MI(VE_PAC,[0 (k/10)],[10 10],[60 60],'no','no','canolty');

end

MI_ozkurt = [];

for k = 1:100 % 0.1-10s in 0.1s steps

MI_ozkurt(k) = calc_MI(VE_PAC,[0 (k/10)],[10 10],[60 60],'no','no','ozkurt');

end

MI_tort = []; % 0.1-10s in 0.1s steps

for k = 1:100

MI_tort(k) = calc_MI(VE_PAC,[0 (k/10)],[10 10],[60 60],'no','no','tort');

end

MI_PLV = []; % 0.1-10s in 0.1s steps

for k = 1:100

MI_PLV(k) = calc_MI(VE_PAC,[0 (k/10)],[10 10],[60 60],'no','no','PLV');

end

xticks = ([0:1:10]);

% Plot results (Canolty)

figure;plot([0.1:0.1:10],MI_canolty,'Color',[0.5 0 0.6],'LineWidth',6);

title('MVL-MI');

xlabel('Trial Length (s)');ylabel('MI Value');

set(gca,'FontName','Arial');

set(gca,'FontSize',30);

set(gca,'XTick',xticks);

% Plot results (Ozkurt)

figure;plot([0.1:0.1:10],MI_ozkurt,'LineWidth',6);

title('MVL-MI');

xlabel('Trial Length (s)');ylabel('MI Value');

set(gca,'FontName','Arial');

set(gca,'FontSize',30);

set(gca,'XTick',xticks);

% Plot Results (Tort)

figure; plot([0.1:0.1:10],MI_tort,'r','LineWidth',6); hold on;

title('KL-MI');

xlabel('Trial Length (s)');ylabel('MI Value');

set(gca,'FontName','Arial');

set(gca,'FontSize',30);

set(gca,'XTick',xticks);

% Plot Results (PLV)

figure; plot([0.1:0.1:10],MI_PLV,'Color',[0 0.7 0.2],'LineWidth',6); hold on;

title('PLV-MI');

xlabel('Trial Length (s)');ylabel('MI Value');

set(gca,'FontName','Arial');

set(gca,'FontSize',30);

set(gca,'XTick',xticks);

%% How does number of bins affect the MI-KL-Tort approach?

tort_PAC_9_bins = calc_MI(VE_PAC,[0.3 1.5],[7 13],[34 100],'no','no','tort',9);

tort_PAC_18_bins = calc_MI(VE_PAC,[0.3 1.5],[7 13],[34 100],'no','no','tort',18);

tort_PAC_36_bins = calc_MI(VE_PAC,[0.3 1.5],[7 13],[34 100],'no','no','tort',36);

% Plot results

figure; xticks = [7:1:13]; subplot(1,3,1);

pcolor([7:1:13],[34:2:100],tort_PAC_9_bins); shading(gca,'interp');

colormap(jet); colorbar;

set(gca,'FontSize',15);

xlabel('Phase Frequency (Hz)','FontSize',25);ylabel('Amplitude Frequency (Hz)','FontSize',25);

title('9 Bins');

set(gca,'FontName','Arial');

set(gca,'XTick',xticks);

subplot(1,3,2);

pcolor([7:1:13],[34:2:100],tort_PAC_18_bins); shading(gca,'interp');

colormap(jet); colorbar;

set(gca,'FontSize',15);

xlabel('Phase Frequency (Hz)','FontSize',25);ylabel('Amplitude Frequency (Hz)','FontSize',25);

title('18 Bins');

set(gca,'FontName','Arial');

set(gca,'XTick',xticks);

subplot(1,3,3);

pcolor([7:1:13],[34:2:100],tort_PAC_36_bins); shading(gca,'interp');

colormap(jet); colorbar;

set(gca,'FontSize',15);

xlabel('Phase Frequency (Hz)','FontSize',25);ylabel('Amplitude Frequency (Hz)','FontSize',25);

title('36 Bins');

set(gca,'FontName','Arial');

set(gca,'XTick',xticks);

set(gcf,'Position',[6 558 1908 420])

**1.8 MATLAB Functions**

%%%%%%%%%%%%%%%%%%%%%%%%%%%%%%%%%%%%%%%%%%%%%%%%%%%%%%%%%%%%%%%%%%%%%%%%%%%

% Function to produce a comodulogram of Phase Amplitude Coupling (PAC)

% Modulation Index (MI) values using the metrics from Tort et al.,(2010),

% Ozkurt & Schnitzler (2011), Canolty et al., (2006) and

% PLV (Cohen 2008).

%

% Inputs:

% - virtsens = MEG data (1 channel)

% - toi = times of interest in seconds e.g. [0.3 1.5]

% - phases of interest e.g. [4 22] currently increasing in 1Hz steps

% - amplitudes of interest e.g. [30 80] currently increasing in 2Hz steps

% - diag = 'yes' or 'no' to turn on or off diagrams during computation

% - surrogates = 'yes' or 'no' to turn on or off surrogates during computation

% - approach = 'tort','ozkurt','canolty','PLV'

% Optional Inputs:

% - Number of phase bins used in KL-MI-Tort approach (default = 18)

%

% Outputs:

% - MI_matrix_raw = phase amplitude comodulogram (no surrogates)

% - MI_matrix_surr = = phase amplitude comodulogram (with surrogates)

%

% For details of the PAC methods go to:

% http://jn.physiology.org/content/104/2/1195.short

% http://science.sciencemag.org/content/313/5793/1626.long

% http://www.sciencedirect.com/science/article/pii/S0165027011004730

% http://www.sciencedirect.com/science/article/pii/S0165027007005237

%

% Written by: Robert Seymour - Aston Brain Centre. July 2017.

%

%%%%%%%%%%%%%%%%%%%%%%%%%%%%%%%%%%%%%%%%%%%%%%%%%%%%%%%%%%%%%%%%%%%%%%%%%%%

function [MI_matrix_raw,MI_matrix_surr] = calc_MI(virtsens,toi,phase,amp,diag,surrogates,approach,varargin)

% Set number of bins used for Tort

if isempty(varargin)

nbin = 18;

else

fprintf('Number of bins set to %s',num2str(varargin{1}))

nbin = varargin{1};

end

if diag == 'no'

disp('NOT producing any images during the computation of MI')

end

% Determine size of final matrix

phase_length = length(phase(1):1:phase(2));

amp_length = length(amp(1):2:amp(2));

% Create matrix to hold comod

MI_matrix_raw = zeros(amp_length,phase_length);

MI_matrix_surr = zeros(amp_length,phase_length);

clear phase_length amp_length

row1 = 1;

row2 = 1;

for phase_freq = phase(1):1:phase(2)

for amp_freq = amp(1):2:amp(2)

%% Bandpass filter individual trials using a two-way Butterworth Filter

% Specifiy bandwith = +- 2.5 * center frequency

Af1 = round(amp_freq -(amp_freq/2.5)); Af2 = round(amp_freq +(amp_freq/2.5));

% Filter data at phase frequency using Butterworth filter

cfg = [];

cfg.showcallinfo = 'no';

cfg.bpfilter = 'yes';

cfg.bpfreq = [phase_freq-1 phase_freq+1]; %+-1Hz - could be changed if necessary

cfg.hilbert = 'angle';

[virtsens_phase] = ft_preprocessing(cfg, virtsens);

% Filter data at amp frequency using Butterworth filter

cfg = [];

cfg.showcallinfo = 'no';

cfg.bpfilter = 'yes';

cfg.bpfreq = [Af1 Af2];

cfg.hilbert = 'abs';

[virtsens_amp] = ft_preprocessing(cfg, virtsens);

% Cut out window of interest - should exlude phase-locked

% responses (e.g. ERPs)

cfg = [];

cfg.toilim = toi; %specfied in function calls

cfg.showcallinfo = 'no';

virtsens_phase_toi = ft_redefinetrial(cfg,virtsens_phase);

virtsens_amp_toi = ft_redefinetrial(cfg,virtsens_amp);

% Variable to hold MI for all trials

MI_all_trials = [];

% For each trial...

for trial_num = 1:length(virtsens.trial)

% Extract phase and amp info using hilbert transform

Phase=virtsens_phase_toi.trial{1, trial_num}; % getting the phase

Amp= virtsens_amp_toi.trial{1, trial_num}; % getting the amplitude envelope

% Switch PAC method based on the approach

switch approach

case 'tort'

[MI] = calc_MI_tort(Phase,Amp,nbin);

case 'ozkurt'

[MI] = calc_MI_ozkurt(Phase,Amp);

case 'canolty'

[MI] = calc_MI_canolty(Phase,Amp);

case 'PLV'

[MI] = calc_MI_PLV(Phase,Amp);

end

% Add the MI value to all other all other values

MI_all_trials(trial_num) = MI;

end

% If user specified to use surrogates - use them!

if strcmp(surrogates, 'yes')

% Variable to surrogate MI

MI_surr = [];

% For each surrogate (surrently hard-coded for 200, could be changed)...

for surr = 1:200

% Get 2 random trial numbers

trial_num = randperm(length(virtsens_phase_toi.trialinfo),2);

% Extract phase and amp info using hilbert transform

% for different trials & shuffle phase

Phase=virtsens_phase_toi.trial{1, trial_num(1)}(randperm(length(virtsens_phase_toi.trial{1,trial_num(1)}))); % getting the phase

Amp = virtsens_amp_toi.trial{1,trial_num(2)};

% Switch PAC approach based on user input

switch approach

case 'tort'

[MI] = calc_MI_tort(Phase,Amp);

case 'ozkurt'

[MI] = calc_MI_ozkurt(Phase,Amp);

case 'canolty'

[MI] = calc_MI_canolty(Phase,Amp);

case 'PLV'

[MI] = calc_MI_PLV(Phase,Amp);

end

% Add this value to all other all other values

MI_surr(surr) = MI;

end

% Calculate average MI over trials

MI_raw = mean(MI_all_trials);

% Subtract the mean of the surrogaates from the actual PAC

% value and add this to the surrogate matrix

MI_surr_normalised = MI_raw-mean(MI_surr);

MI_matrix_surr(row1,row2) = MI_surr_normalised;

end

% Calculate the raw MI score (no surrogates) and add to the matrix

MI_raw = mean(MI_all_trials);

MI_matrix_raw(row1,row2) = MI_raw;

% Show progress of the comodulogram if diag = 'yes'

if strcmp(diag, 'yes')

figure(2)

pcolor(phase(1):1:phase(2),amp(1):2:amp(2),MI_matrix_raw)

colormap(jet)

ylabel('Amplitude (Hz)')

xlabel('Phase (Hz)')

colorbar

drawnow

end

% Go to next Amplitude

row1 = row1 + 1;

(fprintf('Phase: %d Amplitude: %d MI: %d',phase_freq,amp_freq,MI_raw));

end

% Go to next Phase

row1 = 1;

row2 = row2 + 1;

end

%%%%%%%%%%%%%%%%%%%%%%%%%%%%%%%%%%%%%%%%%%%%%%%%%%%%%%%%%%%%%%%%%%%%%%%%%%%

% PAC SUB-FUNCTIONS

%%%%%%%%%%%%%%%%%%%%%%%%%%%%%%%%%%%%%%%%%%%%%%%%%%%%%%%%%%%%%%%%%%%%%%%%%%%

function [MI] = calc_MI_tort(Phase,Amp,nbin)

% Apply Tort et al (2010) approach)

%nbin=18; % % we are breaking 0-360o in 18 bins, ie, each bin has 20o

position=zeros(1,nbin); % this variable will get the beginning (not the center) of each bin

% (in rads)

winsize = 2*pi/nbin;

for j=1:nbin

position(j) = -pi+(j-1)*winsize;

end

% now we compute the mean amplitude in each phase:

MeanAmp=zeros(1,nbin);

for j=1:nbin

I = find(Phase < position(j)+winsize & Phase >= position(j));

MeanAmp(j)=mean(Amp(I));

end

% The center of each bin (for plotting purposes) is

% position+winsize/2

% Plot the result to see if there's any amplitude modulation

if strcmp(diag, 'yes')

bar(10:20:720,[MeanAmp,MeanAmp]/sum(MeanAmp),'phase_freq')

xlim([0 720])

set(gca,'xtick',0:360:720)

xlabel('Phase (Deg)')

ylabel('Amplitude')

end

% Quantify the amount of amp modulation by means of a

% normalized entropy index (Tort et al PNAS 2008):

MI=(log(nbin)-(-sum((MeanAmp/sum(MeanAmp)).*log((MeanAmp/sum(MeanAmp))))))/log(nbin);

end

function [MI] = calc_MI_ozkurt(Phase,Amp)

% Apply the algorithm from Ozkurt et al., (2011)

N = length(Amp);

z = Amp.*exp(1i*Phase); % Get complex valued signal

MI = (1./sqrt(N)) * abs(mean(z)) / sqrt(mean(Amp.*Amp)); % Normalise

end

function [MI] = calc_MI_PLV(Phase,Amp)

% Apply PLV algorith, from Cohen et al., (2008)

amp_phase = angle(hilbert(detrend(Amp))); % Phase of amplitude envelope

MI = abs(mean(exp(1i*(Phase-amp_phase))));

end

function [MI] = calc_MI_canolty(Phase,Amp)

% Apply MVL algorith, from Canolty et al., (2006)

z = Amp.*exp(1i*Phase); % Get complex valued signal

MI = abs(mean(z));

end

end

%%%%%%%%%%%%%%%%%%%%%%%%%%%%%%%%%%%%%%%%%%%%%%%%%%%%%%%%%%%%%%%%%%%%%%%%%%%

%

% check_non_sinusoidal_rise_decay.m

%

% Function to check for non-sinusoidal / sawtooth oscillations by calculating

% the ratio between rise-time and decay time. If ratio is uneven this

% implies that the oscillation is non-sinusoidal/sawtooth-like.

%

% Inputs:

% - virtsens = virtual sensor data from Fieldtrip

% - toi = times of interest (make this shorter than your epoch length to

% avoid edge artefacts

% - phase_freq = phase of interest

%

% Outputs:

% - ratios = array of N trials with ratio between time to peak and time to

% decay

% - time_to_decay_all = times (in ms) of EVERY peak-->trough event

% - time_to_peak_all = times of EVERY trough-->peak event

%

%%%%%%%%%%%%%%%%%%%%%%%%%%%%%%%%%%%%%%%%%%%%%%%%%%%%%%%%%%%%%%%%%%%%%%%%%%%

function [ratios,time_to_decay_all,time_to_peak_all] = check_non_sinusoidal_rise_decay(virtsens,toi,phase)

%

% % Filter data at phase frequency using Butterworth filter

cfg = [];

cfg.showcallinfo = 'no';

cfg.bpfilter = 'yes';

cfg.bpfreq = [phase-1 phase+1]; % Can be widened if necessary

[phase] = ft_preprocessing(cfg, virtsens);

% Extract times of interest

cfg = [];

cfg.toilim = toi;

cfg.showcallinfo = 'no'; %specfied in function calls

phase_toi = ft_redefinetrial(cfg,phase);

% Book-keeping

ratios = zeros(1,length(virtsens.trial)); %variable to hold time_to_decay:time_to_peak for all trials

time_to_decay_all = []; %variable to hold time_to_decay for all trials

time_to_peak_all = [];

p = 1;

%% For every trial number calculate Calculate time to peak and time to decay

for trial_num = 1:length(virtsens.trial)

% Get the time series (for peaks) and flipped time series (for troughs)

trial = phase_toi.trial{1,trial_num}; trialflipped = trial.*-1;

% Find peaks & troughs

[~,peak_locations] = findpeaks(trial);

[~,trough_locations] = findpeaks(trialflipped);

% Equalise the number of peak and trough events

if length(peak_locations) > length(trough_locations)

peak_locations(1) = [];

elseif length(peak_locations) < length(trough_locations)

trough_locations(1) = [];

end

% Calculate time to peak and time to decay

time_to_decay = [];

time_to_peak = [];

if peak_locations(1)<trough_locations(1) %if peak first

for i = 1:length(peak_locations)-1

time_to_decay(i) = trough_locations(i)-peak_locations(i);

time_to_decay_all(p) = trough_locations(i)-peak_locations(i);

time_to_peak(i) = abs(peak_locations(i+1)-trough_locations(i));

time_to_peak_all(p) = abs(peak_locations(i+1)-trough_locations(i));

p = p+1;

end

elseif peak_locations(1)>trough_locations(1) %if trough first

for i = 1:length(peak_locations)-1

time_to_decay(i) = peak_locations(i)-trough_locations(i);

time_to_decay_all(p) = peak_locations(i)-trough_locations(i);

time_to_peak(i) = abs(trough_locations(i+1)-peak_locations(i));

time_to_peak_all(p) = abs(trough_locations(i+1)-peak_locations(i));

p = p+1;

end

end

% Calculate ratio and add to ratios variable

ratios(trial_num) = mean(time_to_decay)./mean(time_to_peak);

end

%%

%%%%%%%%%%%%%%%%%%%%%%%%%%%%%%%%%%%%%%%%%%%%%%%%%%%%%%%%%%%%%%%%%%%%%%%%%%%%

% This function performs statistical analyis on the matrix of modulation

% index (MI) values computed for PAC analysis.

%

% The script loads the comodulogram and adds the necessary info to make

% into a FT data structure.

%

% Group statistics are then computed using cluster-based permutation tests

% based on the Montercarlo method (Maris & Oostenveld, 2007).

%

% Inputs:

% - PAC_name1 = name of the first comdoulogram (specific to my data - can

% be changed)

% - PAC_name2 = name of the second comodulogram (specific to my data - can

% be changed)

% - phase = phase range

% - amp = amplitude range

% - subject = subject list

% - scripts_dir = where the data & scripts are stored

%

% Outputs:

% - stat = statistical output

%

% Written by Robert Seymour (ABC) - January 2017

%%%%%%%%%%%%%%%%%%%%%%%%%%%%%%%%%%%%%%%%%%%%%%%%%%%%%%%%%%%%%%%%%%%%%%%%%%%

function [stat] = get_PAC_stats(PAC_name1,PAC_name2,phase,amp,subject,scripts_dir,surr)

amp_list = [amp(1):2:amp(2)]; phase_list = [phase(1):1:phase(2)];

grandavgA = [];

for i =1:length(subject)

% load PAC1

load([scripts_dir '\' subject{i} '\' PAC_name1]); % non-ICA'd data

% Add FT-related data structure information

MI_post = [];

MI_post.label = {'MI'};

MI_post.dimord = 'chan_freq_time';

MI_post.freq = amp_list;

MI_post.time = phase_list;

% if loading surrogates then the name of the variable is

% matrix_XXX_surrogates

if surr == 1

MI_post.powspctrm = matrix_post_surrogates;

% if loading raw MI estimate then the name of the variable is

% matrix_XXX

else

MI_post.powspctrm = matrix_post;

end

MI_post.powspctrm = reshape(MI_post.powspctrm,[1,length(amp_list),length(phase_list)]);

% Add to meta-matrix

grandavgA{i} = MI_post;

clear matrix_post MI_post

end

grandavgB = [];

% Repeat for matrix_pre

for i =1:length(subject)

% load matrix_pre

load([scripts_dir '\' subject{i} '\' PAC_name2]); % non-ICA'd data

% Add FT-related data structure information

MI_pre = [];

MI_pre.label = {'MI'};

MI_pre.dimord = 'chan_freq_time';

MI_pre.freq = amp_list;

MI_pre.time = phase_list;

% if loading surrogates then the name of the variable is

% matrix_XXX_surrogates

if surr == 1

MI_pre.powspctrm = matrix_pre_surrogates;

% if loading raw MI estimate then the name of the variable is

% matrix_XXX

else

MI_pre.powspctrm = matrix_pre;

end

MI_pre.powspctrm = reshape(MI_pre.powspctrm,[1,length(amp_list),length(phase_list)]);

% Add to meta-matrix

grandavgB{i} = MI_pre;

clear matrix_pre MI_post

end

%% Perform Stats

cfg=[];

cfg.latency = 'all';

cfg.frequency = 'all';

cfg.dim = grandavgA{1}.dimord;

cfg.method = 'montecarlo';

cfg.statistic = 'ft_statfun_depsamplesT';

cfg.parameter = 'powspctrm';

cfg.correctm = 'cluster';

cfg.computecritval = 'yes';

cfg.numrandomization = 1000;

cfg.alpha = 0.05; % Set alpha level

cfg.tail = 0; % Two sided testing

% Design Matrix

nsubj=numel(grandavgA);

cfg.design(1,:) = [1:nsubj 1:nsubj];

cfg.design(2,:) = [ones(1,nsubj) ones(1,nsubj)*2];

cfg.uvar = 1; % row of design matrix that contains unit variable (in this case: subjects)

cfg.ivar = 2; % row of design matrix that contains independent variable (the conditions)

stat = ft_freqstatistics(cfg,grandavgA{:}, grandavgB{:});

%% Compute group difference between matrix_post and matrix_pre

cfg = [];

post_MI = ft_freqgrandaverage(cfg,grandavgA{:})

pre_MI = ft_freqgrandaverage(cfg,grandavgB{:})

cfg = [];

cfg.parameter = 'powspctrm';

cfg.operation = 'subtract';

diff_MI = ft_math(cfg,post_MI,pre_MI)

cfg = [];

cfg.zlim = 'maxabs';

cfg.ylim = amp;

cfg.xlim = phase;

ft_singleplotTFR(cfg,diff_MI); colormap(jet);

%% Display results of stats (very rough - use make_smoothed_comodulograms)

cfg=[];

cfg.parameter = 'stat';

cfg.maskparameter = 'mask';

cfg.maskstyle = 'outline';

cfg.zlim = 'maxabs';

fig = figure;

ft_singleplotTFR(cfg,stat); colormap('jet');

xlabel('Phase (Hz)'); ylabel('Amplitude (Hz)');

end

function make_smoothed_comodulograms(stat, phase, amp)

% Reshape the necessary data

stats_reshaped = squeeze(stat.stat);

mask_reshaped = squeeze(stat.mask);

v = [1];

% Create the figure

figure('color', 'w');

pcolor(phase(1):1:phase(2),amp(1):2:amp(2),stats_reshaped); % colormap

axislim = max(stat.stat(:));

caxis([-axislim axislim]) %threshold

shading interp; colormap(jet);hold on; c =colorbar; %shading, colorbar

contour(phase(1):1:phase(2),amp(1):2:amp(2),mask_reshaped,v,'--','Color','black','LineWidth',3) %stats mask

set(gca,'FontSize',30);

ylabel('Amplitude Frequency (Hz)','FontSize',25); xlabel('Phase Frequency (Hz)','FontSize',25) %axis labels

ylabel(c,'t-value','FontSize',25);

set(gca,'FontName','Arial');

set(gcf, 'Color', 'w');

set(gca,'XTick',[phase(1):1:phase(2)]);

end

%%%%%%%%%%%%%%%%%%%%%%%%%%%%%%%%%%%%%%%%%%%%%%%%%%%%%%%%%%%%%%%%%%%%%%%%%%%

%

% synthesize_pac.m

%

% Function to simulate a signal containing phase-amplitude coupling

% between the phase of a low frequency (10-11Hz Hz) and amplitude of a

% high-freqency band (50-70 Hz) for a chosen noise level. Hamming tapered

% high frequency signal is added at each cycle of low frequency component.

% sampling frequency is chosen as 1000 Hz.

% Code is adapted from Kramer et al. (2008), Jrn. Nrsc. Methds.

% and Ozkurt et al., (2011) Jrn. Nrsc. Methds.

% Inputs:

% - noise lev : parameter describing the noise power

% Outputs:

% - snr : signal-to-noise ratio

% - s_final: the synthesized signal

%%%%%%%%%%%%%%%%%%%%%%%%%%%%%%%%%%%%%%%%%%%%%%%%%%%%%%%%%%%%%%%%%%%%%%%%%%%

function [s_final, snr] = synthesize_pac(noise_lev)

dt = 0.001;

s = [];

for k=1:400

f = rand()*1.0+10.0; %Create the low freq (10 Hz) signal.

s1 = cos(2.0*pi*(0:dt*f:1-dt*f));

good = find(s1 < -0.99);

s2 = zeros(1,length(s1));

stemp = randn(1,3000); %Create noisy data.

stemp = ft_preproc_bandpassfilter(stemp, 1000, [50 70]);%Make high freq (50-70 Hz) signal.

stemp = stemp(2000:2039); %Duration 50 ms.

stemp = 5*hanning(40)'.*stemp; %Hanning tapered.

rindex = ceil(rand()*2); %Add the high frequency in,

%when the low frequency is near 0 phase.

s2(rindex + good(1) - 20:rindex+good(1)+20-1)=stemp;

s = [s, s1+1*s2];

end

% Generate noise and calculate SNR

n = noise_lev*randn(1,length(s));

snr = 10 * log10((s*s') / (n*n'));

% Add in noise

s = s + n;

s = s(1:12000);

sCropped = s(1000:11000-1);

s_final = sCropped;

**1.1.9 sensory_PAC.m – modify this script to match the paths on your PC**

data_dir = 'D:\sensory_PAC_data';

scripts_dir = 'D:\scripts\sensory_PAC';

fieldtrip_dir = 'D:\fieldtrip-20161024';

subject = {'sub-01','sub-02','sub-03','sub-04','sub-05','sub-06','sub-07',...

'sub-08','sub-09','sub-10','sub-11','sub-12','sub-13','sub-14',...

'sub-15','sub-16'};

1. **Supplementary Data – Indices of Rejected Trials**

| participant_id | trials_rejected |
| --- | --- |
| sub-01 | [] |
| sub-02 | [1] |
| sub-03 | [] |
| sub-04 | [] |
| sub-05 | [] |
| sub-06 | [] |
| sub-07 | [4,46,58,63] |
| sub-08 | [] |
| sub-09 | [] |
| sub-10 | [51] |
| sub-11 | [] |
| sub-12 | [] |
| sub-13 | [] |
| sub-14 | [] |
| sub-15 | [42] |
| sub-16 | [30] |

# Supplementary Analysis – The influence of number of phase bins on KL-MI-Tort phase-amplitude coupling estimation


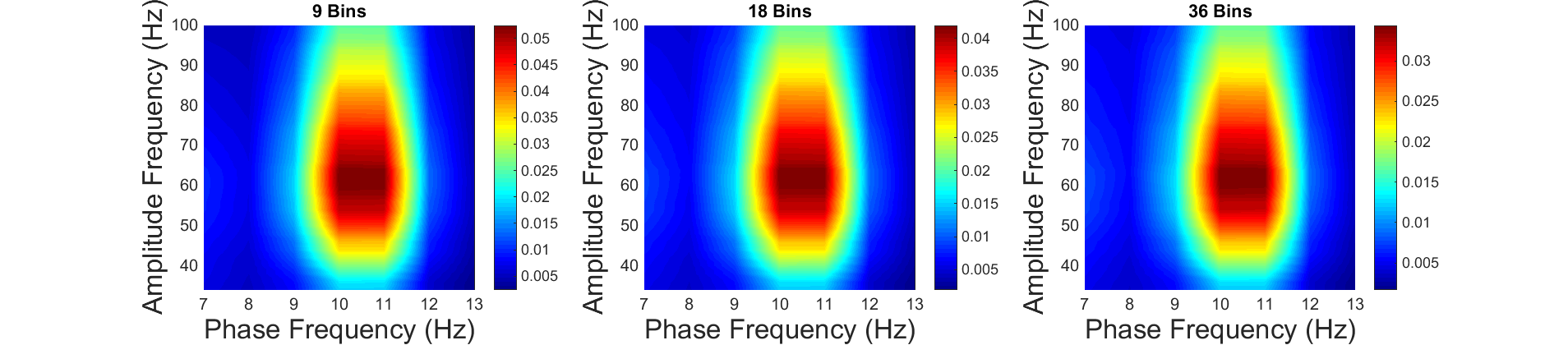


*Figure S1.* Whilst many researchers choose 18 phase bins to compute PAC using the KL-MI-Tort approach, this number is arbitrary. We therefore altered the numbers of phase bins (9, 18 and 36 phase bins) used in KL-MI-Tort computation, and investigated the effect on the ability to detect simulated PAC between 10-11Hz phase and 50-70Hz amplitude. All three phase bins were able to detect the alpha-gamma PAC, with no clear differences in the estimates of modulating phase or amplitude.

Script: 7_simulated_PAC_analysis.m
